# Impact of SNR, peripheral auditory sensitivity, and central cognitive profile on the psychometric relation between pupillary response and speech performance in CI users

**DOI:** 10.3389/fnins.2023.1307777

**Published:** 2023-12-21

**Authors:** Yue Zhang, M. Amparo Callejón-Leblic, Ana M. Picazo-Reina, Sergio Blanco-Trejo, François Patou, Serafín Sánchez-Gómez

**Affiliations:** ^1^Department of Research and Technology, Oticon Medical, Vallauris, France; ^2^Oticon Medical, Madrid, Spain; ^3^ENT Department, Virgen Macarena University Hospital, Seville, Spain; ^4^Biomedical Engineering Group, University of Sevillel, Sevillel, Spain; ^5^Department of Research and Technology, Oticon Medical, Smørum, Denmark

**Keywords:** CI, listening effort, pupillometry, cognitive abilities, speech performance

## Abstract

Despite substantial technical advances and wider clinical use, cochlear implant (CI) users continue to report high and elevated listening effort especially under challenging noisy conditions. Among all the objective measures to quantify listening effort, pupillometry is one of the most widely used and robust physiological measures. Previous studies with normally hearing (NH) and hearing-impaired (HI) listeners have shown that the relation between speech performance in noise and listening effort (as measured by peak pupil dilation) is not linear and exhibits an inverted-U shape. However, it is unclear whether the same psychometric relation exists in CI users, and whether individual differences in auditory sensitivity and central cognitive capacity affect this relation. Therefore, we recruited 17 post-lingually deaf CI adults to perform speech-in-noise tasks from 0 to 20 dB SNR with a 4 dB step size. Simultaneously, their pupillary responses and self-reported subjective effort were recorded. To characterize top-down and bottom-up individual variabilities, a spectro-temporal modulation task and a set of cognitive abilities were measured. Clinical word recognition in quiet and Quality of Life (QoL) were also collected. Results showed that at a group level, an inverted-U shape psychometric curve between task difficulty (SNR) and peak pupil dilation (PPD) was not observed. Individual shape of the psychometric curve was significantly associated with some individual factors: CI users with higher clinical word and speech-in-noise recognition showed a quadratic decrease of PPD over increasing SNRs; CI users with better non-verbal intelligence and lower QoL showed smaller average PPD. To summarize, individual differences in CI users had a significant impact on the psychometric relation between pupillary response and task difficulty, hence affecting the interpretation of pupillary response as listening effort (or engagement) at different task difficulty levels. Future research and clinical applications should further characterize the possible effects of individual factors (such as motivation or engagement) in modulating CI users’ occurrence of ‘tipping point’ on their psychometric functions, and develop an individualized method for reliably quantifying listening effort using pupillometry.

## 1 Introduction

Cochlear implantation is a standard care for individuals with severe-to-profound sensorineural hearing loss who obtain limited benefit from conventional hearing aids. Typically, cochlear implant (CI) recipients show good levels of speech communication in quiet, but the high inter-subject variability observed in outcome measures remains a challenge in both clinical and research fields (Lazard et al., 2012; Holden et al., 2013; Boisvert et al., 2020; Goudey et al., 2021). Specifically, CI users find it difficult to listen in complex daily-life situations and noisy environments. Such variability may be due to many factors including, but not limited to, the age of implantation, duration of deafness, duration of CI use, the limited spectral and temporal resolution of the signal, quality of the electrode-neural interface, and the narrow electric dynamic range of the output signals codified at the stimulation electrodes (Blamey et al., 2012; Holden et al., 2013; Dorman and Gifford, 2017; Boisvert et al., 2020). CI users often complain of the high listening effort required in those situations, even when speech communication is successful (Hughes and Galvin, 2013; Winn et al., 2015; Pichora-Fuller et al., 2016; Winn, 2016; Rapport et al., 2020; Dingemanse and Goedegebure, 2022; Winn and Teece, 2022). Eventually, this sustained and unresolved high effort status associated with hearing could have negative consequence on social communication and cognitive health for CI users (Nachtegaal et al., 2009; Pichora-Fuller et al., 2015; Herrmann and Johnsrude, 2020; Shukla et al., 2020).

Although speech recognition is a conventional metric of CI performance, it does not capture the listening effort experienced by CI users when they expend cognitive resources to achieve a certain level of speech performance. Quantifying listening effort provides an additional layer of information to complement and inform the efficiency of speech intelligibility measures. Different methods to quantify listening effort have been proposed, including physiological, subjective, and behavioral methods. Together, these methods reveal different underlying domains of listening effort (Strand et al., 2018; Shields et al., 2023). Specifically, pupillometry is one of the most widely used physiological methods. Pupillometry has been proved to be a robust technique in registering changes in cognitive effort (Kramer et al., 1997; Zekveld and Kramer, 2014; Zekveld et al., 2018). Different studies have demonstrated that pupillometry measures can capture the intensity of effort, and it has been already documented to be sensitive to speech intelligibility, spectral resolution, linguistic complexity, attention, concurrent cognitive demands, background noise, etc. (Koelewijn et al., 2012, 2014; Besser et al., 2013; Winn et al., 2015; Ohlenforst et al., 2017; Zekveld et al., 2019; Bönitz et al., 2021; Micula et al., 2021; Zhang et al., 2021). Pupillometry also holds great potential to be adapted into the clinical setting due to its non-invasive nature and its robustness against electrical CI artifacts.

Benefits and limitations of CI on speech recognition have been extensively researched. Relatively fewer studies have measured listening effort in CI users, even though the findings from the limited studies all suggest the importance of including listening effort measures when assessing CI outcomes. One of the difficulties preventing more listening effort studies (specifically using pupillometry), both in research and clinics, is our lack of understanding of the psychometric relation between CI users’ speech performance and listening effort measured by pupillary response. As shown by previous studies (Ohlenforst et al., 2017, 2018; Wendt et al., 2017), the relation between speech performance in noise and listening effort (as measured by peak pupil response) is not linear in normal hearing (NH) and hearing-impaired (HI) participants: listeners show bigger peak pupil response (PPD), one of the most widely used pupillary marker for listening effort, as the sentence recognition in noise task gets more difficult, until they reach a certain “tipping point” and disengage from the task as it gets overly difficult. From there onwards, listeners show smaller PPD as the task increases in difficulty. On a group level, the psychometric relation between task difficulty and pupillary response showed an inverted U-shape: in high SNR regions, higher SNR relates to lower PPD; and in low SNR regions higher SNR relates to bigger PPD. Many studies have used this psychometric relation as the basis to interpret changes in pupil response as the effectiveness of intervening strategies in reducing listening effort for NH and HI listeners (Winn, 2016; Ohlenforst et al., 2018). However, no study has yet validated this relation for CI users across a similarly wide range of listening conditions (i.e., 9 SNR steps with speech recognition score ranging from 0 to 100%). It is highly likely that CI users do not have similar hearing and physiological status as NH and HI listeners, thus CI users’ psychometric relation between listening task and pupillary response might not be the same as in NH and HI listeners (Hughes and Galvin, 2013; Wang et al., 2016; Winn, 2016; Perreau et al., 2017; Perea Pérez et al., 2023). Therefore, without such validation with CI users on this piece of evidence, it is difficult to establish enough confidence to use pupillometry to interpret listening effort and assess efficacy of new CI interventions, such as new signal processing strategies, noise reduction and beamforming algorithms, among others.

Furthermore, past studies have investigated the relation between CI speech outcomes and individual auditory and cognitive factors. But it is unclear whether and how individual differences in auditory sensitivity and central cognitive resources affect listening effort and the psychometric relation between listening conditions and pupillary responses in CI users. It is unlikely that all CI users would bear the same psychometric relations, considering the big variabilities in CI outcomes. Therefore, it is important to identify and quantify the effects of individual factors that contribute to the variabilities in the psychometric relation. Multiple studies have shown significant correlation between spectral-temporal acuity with speech performance, suggesting that bottom-up auditory processing ability is important to explain CI variabilities (Aronoff and Landsberger, 2013; Lawler et al., 2017; DiNino and Arenberg, 2018). In addition to biological and audiological factors, individual cognitive capacities, i.e., top-down abilities, have been shown to affct both the speech and listening effort outcomes in CI users (Pisoni, 2000; Amini et al., 2023; Beckers et al., 2023). Working memory (WM) is a factor of most interest due to its association with speech performance in pediatric and adult CI users, HI and old NH listeners (Pisoni and Cleary, 2003; Akeroyd, 2008; Rudner et al., 2011; Besser et al., 2013; Füllgrabe and Rosen, 2016; Kestens et al., 2021; Dingemanse and Goedegebure, 2022). WM is a limited-capacity system with simultaneous storage and processing mechanisms whereby encoded information is analyzed and manipulated (Daneman and Carpenter, 1980). Studies using WM tasks incorporating either the processing speed or efficiency (for instance forward-and-backward digit span, reaction speed etc.) showed significant relation with CI users’ speech recognition (Tao et al., 2014; Moberly et al., 2017a,b; Völter et al., 2021). Nonverbal reasoning tests have also been suggested as a method to predict speech recognition in CI users, because the ability to solve new tasks using limited information should, intuitively, relate to the ability to fuse degraded auditory inputs into a meaningful percept. Past studies have reported a significant correlation between CI users’ sentence recognition and their non-verbal reasoning skills, although the underlying relation between nonverbal reasoning and processing efficiency remains unclear (Carpenter et al., 1990; Mattingly et al., 2018; Moberly et al., 2019; Beckers et al., 2023). Cognitive inhibition is the ability to suppress distracting information, and past studies have shown that this ability could be linked with CI word and sentence recognition (Moberly et al., 2018; Tamati et al., 2020; Völter et al., 2021; Tamati and Moberly, 2022). Considering the widely reported impact of individual cognitive abilities on CI outcomes, it is important to understand how CI users’ individual cognitive capacities affect the relation between speech performance and listening effort, to choose the most optimal test levels for each CI user.

Therefore, the present study aims to, firstly, investigate the non-linear relationship between pupil response and speech performance in noisy conditions for CI users. Speech recognition and listening effort (measured by both concurrent pupillary response and subjective ratings) were tested in a wide range of SNR levels across the entire psychometric function of CI users, similarly to (Ohlenforst et al., 2018). We hypothesize that a similar inverse U-shape relation between speech performance and pupillary response would appear consistently in CI users, albeit with higher individual variability compared to NH and HI listeners.

The second aim of the present study is to identify sources and patterns in CI users’ profiles that allow us to explain individual variabilities in the non-linear relationship mentioned above. The present study evaluates specifically the effect of neurocognitive factors (i.e., top-down) and auditory sensitivity (i.e., bottom-up), as measured by visual word-color (Stroop, 1935) (i.e., cognitive inhibition), N-back (Gevins and Cutillo, 1993) (i.e., working memory), Progressive Matrices (Harris et al., 2020) (i.e., non-verbal intelligence), and Spectral Modulation Ripple Test (SMRT) (Aronoff and Landsberger, 2013) (i.e., spectral-temporal sensitivity). We hypothesize that individual differences in CI users’ performance and top-down/bottom-up profiles predict the shape of the psychometric relation between SNR and pupillary response.

To sum up, results of the present study will enrich our knowledge on the variable outcomes of speech perception and listening effort in CI users. It will help future research and clinics to interpret better the pupillometry outcomes when applying this method to quantify listening effort in CI users and assess innovative CI strategies.

## 2 Materials and methods

This section describes: (1) the characteristics of the recruited patients and (2) the experimental design and measures collected, which include: (a) clinical fitting and audiometry testing, (b) a hearing in noise test (HINT) as auditory stimuli to elicit pupil responses, (c) a setup for pupillometry recording to objectively quantify listening effort; (d) self-reported measures of subjective effort and cognitive load; (e) a spectro-temporal resolution modulation test; (f) cognitive tests to evaluate working memory, inhibitory control and non-verbal intelligence, and (g) quality of life. Finally, (3) data pre-processing and statistical methods are described.

### 2.1 Participants

Seventeen subjects (10 females and 7 males) with a mean age of 53.8 ± 12.2 years (range 36–69 years) volunteered to participate in the study from January to December 2022. All participants had been diagnosed with post-lingual severe-to-profound hearing loss and were unilateral Neuro Zti (Oticon Medical®) users with at least 12 months of experience with their CI (40.2 ± 11.1, range 12–59 months). Further demographic and clinical data are listed in Table 1. All the participants had normal or corrected-to-normal vision and no history of neurological or psychiatric diseases. The study was approved by the Ethics Committee of the Hospital Universitario Virgen Macarena in Seville, Spain (PEIBA#19/2019). All participants have written informed consent.

**Table 1:** Demographic information of CI participants.

Participant	Age	Sex	Ear	Implant type	Array	Time of use (months)	Etiology
1	65	M	RIGHT	Neuro Zti	EVO	32	Unknown
2	60	F	LEFT	Neuro Zti	EVO	35	Sudden HL
3	44	F	RIGHT	Neuro Zti	EVO	42	Unknown
4	65	F	LEFT	Neuro Zti	EVO	45	Ototoxic
5	64	M	LEFT	Neuro Zti	EVO	32	Unknown
6	69	F	LEFT	Neuro Zti	EVO	41	Unknown
7	45	M	LEFT	Neuro Zti	EVO	32	Possible hepatitis
8	52	F	RIGHT	Neuro Zti	EVO	37	Hepatitis disease
9	39	F	LEFT	Neuro Zti	EVO	35	Unknown
10	45	M	LEFT	Neuro Zti	EVO	50	Unknown
11	61	M	LEFT	Neuro Zti	EVO	51	Unknown
12	53	M	RIGHT	Neuro Zti	EVO	42	Head trauma
13	59	F	RIGHT	Neuro Zti	EVO	39	Unknown
14	27	F	RIGHT	Neuro Zti	EVO	12	Unknown
15	63	M	LEFT	Neuro Zti	EVO	58	Unknown
16	48	F	RIGHT	Neuro Zti	EVO	59	Meniere disease
17	36	F	RIGHT	Neuro Zti	EVO	42	Unknown

### 2.2 Experimental procedure

#### 2.2.1 Clinical fitting and testing

Electrode impedances and maximum comfortable C levels were checked prior to the experimental speech test and pupillometry recording using the CI-link interface and the Genie Medical software (Oticon Medical®). All the participants wore a Neuro 2 sound processor with their most-frequently used fitting map. The noise reduction setting was deactivated to control behaviors of the automatic noise cancelation algorithm in laboratory conditions (Guevara et al., 2016). The compression system was set to the static version to avoid variable compression performance across the wide range of SNR conditions (Bozorg-Grayeli et al., 2016). Subsequently, participants were subjected to free-field aided audiometry from 250 Hz to 8,000 Hz with narrow-band stimuli (for detailed analysis on CI users’ audiometry tested, see Supplementary Material 1). Finally, their word recognition was evaluated in quiet through a list of 25 Spanish disyllabic words (de Cárdenas and Marrero, 1994).

#### 2.2.2 Stimuli

Auditory stimuli were Castilian Spanish sentences from the Hearing in Noise Sentence Test (HINT), embedded in speech-shaped noise matching the long-term spectrum of the sentence materials. Although the present study aims to target the complete psychometric range as in Ohlenforst et al. (2017, 2018), only a subset of SNRs was chosen because typical daily listening scenarios have SNRs ranging from 5 to 15 dB SNR (Smeds et al., 2015) and CI users show different ranges and slopes of their speech psychometric functions (Bergeron and Hotton, 2016; Cordeiro et al., 2021). CI users also experience near-floor performance in negative SNRs, therefore not useful to inform on the psychometric shape of intelligibility and effort. Therefore, the present study tested 6 steps from 0 to +20 dB SNR in increment of 4 dB SNR: 0 dB, 4 dB, 8 dB, 12 dB, 16 dB, 20 dB SNR.

All stimuli were presented through a frontal loudspeaker located at 0° azimuth from the participant, who was sat on a chair located approximately 120 cm ±3 cm from the speaker in a soundproof room. Noise levels for all SNR conditions were fixed at 65 dB SPL, and speech levels varied accordingly. Note that due to the wide range of SNR tested in the current study, prior to the experiment, a quick test was administered to ensure that speech level at 0 dB SNR was audible, and overall loudness at 20 dB SNR was loud but tolerable. CI users were presented with an initial training list of 20 sentences at a maximum SNR of +20 dB SNR. Subsequently, different lists of 20 sentences were presented, twice per different SNR conditions. The presentation of the SNR conditions was randomized. Overall, 240 sentences (40 sentences per 6 SNR conditions) were tested per participant.

#### 2.2.3 Concurrent pupillometry

Pupil diameter was recorded using an infrared eye tracking system PupilLabs Core^1^ (Kassner et al., 2014). Pupil is an open-source platform for mobile eye-tracking and gaze-based measurements. The experimenter monitored and ensured the quality and stability of the real-time pupil responses recorded before and during the session, as well as other settings such as the camera exposure and intensity parameters available in the eye-tracker Pupil GUI software. Participants were instructed to fixate their eyes on a fixation cross at the TV screen and were asked not to move or blink brusquely as much as possible. Room luminance was controlled by adjusting the room ambience light and screen brightness to reach an average value around 55 ± 5.1 lux, using a luxmeter positioned at the eye level of the participant facing the screen. The luminance level was then kept stable throughout the recording to ensure no impact on pupil traces from non-task-evoked pupillary response (Zhang et al., 2022). The eye-tracker device was connected to a laptop and the pupil data was collected through customized Python scripts developed within the OMEXP research platform (Sulas et al., 2022).

In each trial, each participant was instructed to fixate on a light gray fixation cross (+) on the dark gray screen. The background noise started 2 s before the sentence’s onset, serving as a baseline measurement. The background noise then ended 2 s after the offset of the sentence, allowing for task-evoked pupillary response to peak (Winn et al., 2018). After the offset of background noise, the fixation cross changed to a cross (X), indicating the participant to repeat the sentence aloud. The experimenter noted down the correctly repeated keywords in the sentence then moved on to the next trial. Pupil recordings were synchronized with HINT sentences at interval points corresponding to the start of each trial (noise onset), sentence onset and post-noise offset using the Lab Streaming Layer (LSL) protocol (Kothe et al., 2012). To avoid fatigue in some patients, the experiment was run in two different experimental sessions on two separate days (at around the same time of the day) if needed. Note that past studies have shown that task performance and pupil responses can be situationally dependent and can vary by the day (Veneman et al., 2013; Winn et al., 2018). However, as shown later in the analysis section, the current experiment corrected pupillary response by the trial baseline which could alleviate the day-to-day variation in absolute pupil diameter. Therefore, to control for the confounding effect of fatigue on task-evoked pupil responses, the test organization was prioritized to avoid fatigue (McGarrigle et al., 2017; Zekveld et al., 2018).

#### 2.2.4 Subjective measures of mental workload and effort

After each SNR condition (20 sentences), subjects were asked to report their subjective mental workload using a computer version of the well-known NASA Task Load Index (TLX) (Hart and Staveland, 1988). This yields a multimodal overall workload score calculated from a weighted average of subscale ratings including six categories: physical demand, temporal demand, mental demand, self-performance, effort, and frustration. The test comprised two phases: 1) from two categories, the patient selected the one that best reflected the sensation experienced during the task. All combinations of two categories were tested; 2) for each category, a visual analog scale ranging from “low” to “high” or from “good” to “poor” (in the case of performance) was presented to the user. Scores were calculated following the standard procedure.

#### 2.2.5 Spectro-temporal resolution

To quantify spectral resolution, the SMRT (Aronoff and Landsberger, 2013) was administered with the same setting conditions, at 65 dB SPL from a frontal loudspeaker located at 0° azimuth from the participant. In each trial, participants were presented with three stimuli (2 references; 1 target) and were asked to press a button to indicate which of the three stimuli sounded different. The references were fixed at 20 ripples per octave (RPO). The target initially had a ripple density of 0.5 RPO. The ripple density of the target varied adaptatively depending on user responses using a one-up/one-down procedure and a step size of 0.2 RPO. The test stopped after ten reversals, and the last six reversals were averaged to give an overall SMRT threshold (in RPO) for each participant.

#### 2.2.6 Neurocognitive functioning

Working memory was assessed by the visual N-back test with N = 2 which involves a sequence of alphabets presented in a computer screen. In the 2-back test, subjects were asked to press a key on a keyboard each time an alphabet shown on the screen was identical to that shown two alphabets before. Each run comprised 22 alphabets and each participant took two runs. Discriminability d-prime was calculated using R package psycho (Makowski, 2018), as an index of working memory efficiency (Haatveit et al., 2010).

Inhibitory control was assessed using Stroop color-word visual test based on the original version (Stroop, 1935). During each trial, a color word was presented on a computer screen, presented in a specific font color, and the participant was asked to press a key on a keyboard corresponding to the font color and not the name of the color word. The test contains three distinct types of trials: congruent trials (color font and color word match), incongruent trials (color font and color word do not match), and control trials (a rectangle of a given color is shown). Each type was blocked and comprised of 12 unique trials. Each block was repeated 3 times, and the block sequence was randomized. Stroop color-word interference scores were calculated by subtracting the mean correct of the incongruent trials by the congruent trials. The average reaction time in control trials was also calculated for later analysis.

Lastly, nonverbal intelligence was measured using a computer version of Progressive Matrices, which shows a series of progressive 3 × 3 visual grid patterns that become increasingly difficult in time (Matzen et al., 2010; Harris et al., 2020). Participants were asked to complete the pattern by selecting the last unfilled grid from several options shown. Participants were scored based on how many items they correctly completed.

This set of neurocognitive tests intended to capture the differences in CI users’ abilities. N-back task assessed CI users’ abilities to store and process the activated phonological and lexical competitors from the ambiguous inputs; Stroop color-word test assessed their abilities to reject incorrect candidates; and Progressive Matrices task assessed their abilities to fuse ambiguous information into a complete percept.

The spectro-temporal and neurocognitive tests were also performed using the OMEXP computer platform based on custom-made and validated Python scripts (Sulas et al., 2022).

#### 2.2.7 Quality of life

Quality of life (QoL) was evaluated through the Nijmegen Cochlear Implant Questionnaire (NCIQ) (Hinderink et al., 2000). NCIQ is a well-known and commonly used test to evaluate auditory QoL in CI users. NCIQ comprises three general domains (physical, psychological, and social), which can be divided into six different subdomains: the physical domain is formed by three subdomains: basic sound perception (NCIQ1), advanced sound perception (NCIQ2) and speech production (NCIQ3). The psychological domain is formed by the subdomain self-esteem (NCIQ4), and the social domain comprises two subdomains; activity (NCIQ) and social interactions (NCIQ6). Each of these subdomains comprises ten questions or statements answered through a five-point response scale ranging from ‘never’ to ‘always’ or from ‘no’ to ‘good’. If the question does not apply to a patient, a sixth answer ‘not applicable’ is also available. Final overall scores range from 0 (very poor) to 100 (optimal) for each subdomain and globally.

### 2.3 Data pre-processing and analysis

#### 2.3.1 Pupil data pre-processing

Pupil traces collected from the left-eye camera were pre-processed to detect eye blinks and abnormalities. Any sample point with a value below or above 3 Median Absolute Deviation (MAD) of the median pupil diameter of the recording was counted as blinks or outliers and interpolated using cubic interpolation (Leys et al., 2013). Additionally, sample points 35 ms before and 100 ms after the abnormality were also discarded to avoid blink onset and offset effects. The missing samples were cubically interpolated based on the preserved samples of the trace and smoothed with a running average filter with 100-ms duration. Trials with over 40% of the sample points interpolated were excluded from further analysis. A previous study also supported that 45% blink exclusion criterion would not affect group pupil results compared to more stringent criteria (Burg et al., 2021). On this basis, 12% of all trials were excluded, and no participant was excluded because no participant had over 30% trials excluded.

The baseline pupil diameter in each trial was calculated as mean pupil diameter averaged over 2 s before the onset of the sentence. The pupil diameter measured from the sentence onset to the repeat prompt was subtracted from that baseline level and then normalized by the baseline diameter to obtain the relative pupil diameter changes elicited by the task, according to the formula below (Wagner et al., 2019):


(1) $$\frac{\mathrm{observation}-\mathrm{baseline}}{\mathrm{baseline}}\;\times 100$$

Processed traces were then aggregated per participant and per SNR condition, aligned by the onset of the sentences. The search for the maximum pupil dilation was restricted to the time window starting from sentence onset and ending at the verbal response prompt (thus excluding any possible confounding effect with the pupil arousal induced when verbally repeating the sentence), and the maximum dilations were recorded as PPD for later analyses. Note that all sentences were left unprocessed in duration to avoid unnatural acoustic manipulation (compression or stretching of original sentences). This procedure was common in listening effort studies due to varied length of standardized sentences and the variability in sentence duration could be well controlled by consistent trace alignment (either by sentence onset or offset). All pupil data pre-processing was conducted on Matlab 2016b using custom-made scripts.

#### 2.3.2 Statistical analysis

To examine the first hypothesis that a consistent psychometric relation similar to that of NH and HI listeners exists for CI users, a mixed-effect linear regression model was built, using PPD as dependent variable, SNR condition as the fixed effect factor, and participant as random effect factor. The model was constructed using the lme4 package (Bates et al., 2015) in R and figures were produced using the ggplot2 package (Wickham, 2016). Fixed and random effect factors entered the model, and remained in the model only if they significantly improved the model fitting, using Chi-squared tests based on changes in deviance (*p* < 0.05). Differences among levels of SNR condition were examined using post-hoc Wald test, and *p* values were estimated using the z distribution in the test as an approximation for the t distribution (Mirman, 2017). To perform sanity checks on whether behavioral performance and subjective rating in speech-in-noise tasks were consistent with past CI results, two mixed-effect logistic regression models were built, using keywords proportion correct and NASA-TLX scores as dependent variables respectively, SNR condition as the fixed effect factor and participant as random effect factor.

Considering that we hypothesized an inverted-U shape for the psychometric relation between task difficulty and pupillary response, a mixed-effect linear regression model with linear and quadratic polynomial terms was constructed. The model used PPD as dependent variable, linear and quadratic term of SNR condition as fixed effect factors and participant as random effect factor, to examine whether linear (i.e., rising or falling) and quadratic trends (i.e., U-shape or inverse U-shape) existed from 0 dB SNR to 20 dB SNR. Model selection and *post-hoc* tests were performed in the same way as above.

To examine the second hypothesis that individual variability could predict the shape of the psychometric relation, a series of correlation tests were performed. For parameters relating to the shape of the psychometric relation, average PPD and quadratic coefficients of the polynomial fitting of PPD from SNR0 to SNR20 were calculated for each participant. For measured individual differences in CI performance and top-down/bottom-up factors, age, CI duration, clinical word recognition in quiet, auditory thresholds measured in free field (T-level), QoL, sentence keyword corrects in noise, NASA-TLX rating in noise, Progressive Matrices correct, Stroop inference scores in correct, Stroop control reaction time, N-back d-prime and SMRT threshold were considered. Pairwise correlation tests were performed to examine the strength of correlation between individual differences and psychometric shape parameters. Pearson’s r was used when two factors under comparison were continuous, and Spearman’s rho was used when either factor was non-continuous (for instance, values bounded between 0 and 100%) or non-normal bivariate distribution. Bonferroni correction on *p*-values was applied to correct for multiple testing.

## 3 Results

### 3.1 Sentence recognition and subjective effort rating

A significant effect of SNR condition on sentence keyword recognition score was found (*χ*^2^ = 118.3, df = 5, *p* < 0.001). Post-hoc z-tests showed a consistent trend that higher SNRs were related to better recognition scores. Statistically significant comparisons are: SNR12 > SNR4 (β = 4.33, SE = 1.12, *p* < 0.001), SNR16 > SNR4 (β = 6.41, SE = 1.53, *p* < 0.001), SNR20 > SNR4 (β = 6.96, SE = 1.61, *p* < 0.001), SNR12 > SNR8 (β = 2.81, SE = 1.01, *p* = 0.005), SNR16 > SNR8 (β = 4.89, SE = 1.4, *p* < 0.001), SNR20 > SNR8 (β = 5.43, SE = 1.49, *p* < 0.001), SNR20 > SNR12 (β = 2.63, SE = 1.33, *p* = 0.03).

**Figure 1:**
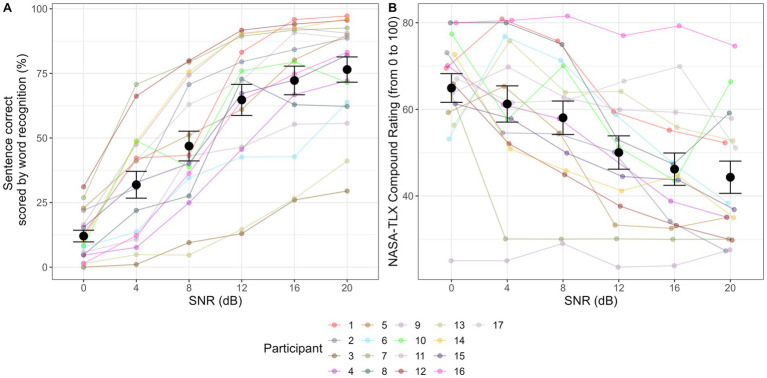
Sentence keyword correct and NASA-TLX rating in sentence recognition in noise tasks. Panel **(A)** shows keyword correct at 0 dB, 4 dB, 8 dB, 12 dB, 16 dB, and 20 dB SNR conditions. Panel **(B)** shows NASA-TLX rating at 0 dB, 4 dB, 8 dB, 12 dB, 16 dB, and 20 dB SNR conditions. Each colored dot corresponds to the average performance of each participant. Black dot corresponds to the average performance across 17 participants, and the error bar corresponds to 1 standard error (SE) from the mean.

A significant effect of SNR condition on NASA-TLX rating was found (*χ*^2^ = 21.62, df = 5, *p* < 0.001). Post-hoc z-tests showed a consistent trend that higher SNRs were related to easier ratings. Statistically significant comparisons are: SNR12 < SNR0 (β = −0.81, SE = 0.26, *p* = 0.002), SNR16 < SNR0 (β = −0.81, SE = 0.26, *p* = 0.002), SNR20 < SNR0 (β = −0.86, SE = 0.26, *p* < 0.001), SNR12 < SNR4 (β = −0.53, SE = 0.26, *p* = 0.04), SNR16 < SNR4 (β = −0.53, SE = 0.25, *p* = 0.04), SNR20 < SNR4 (β = −0.58, SE = 0.25, *p* = 0.02), SNR12 < SNR8 (β = −0.59, SE = 0.25, *p* = 0.02), SNR16 < SNR8 (β = −0.58, SE = 0.25, *p* = 0.02), SNR20 < SNR8 (β = −0.64, SE = 0.25, *p* = 0.01). Figure 1 shows the results of sentence keyword recognition (Figure 1A) and NASA-TLX score (Figure 1B).

### 3.2 PPD and psychometric relation

**Figure 2:**
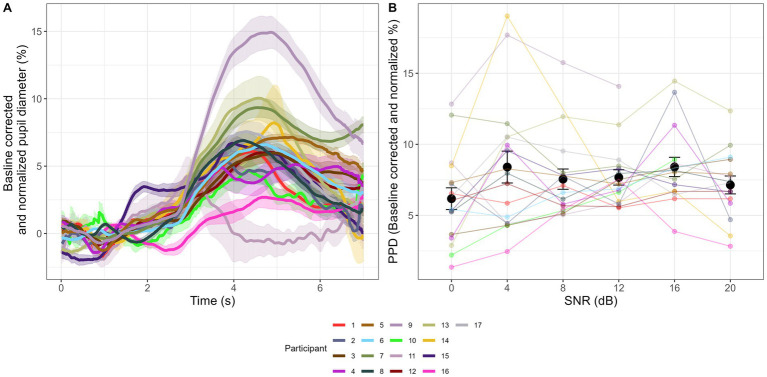
Pupillometry results in sentence recognition in noise tasks. Colored lines in Panel **(A)** indicate aggregated pupil traces of each participant and shaded areas show 1 standard error (SE) region from the mean. 0 s – 2 s is the baseline period where participant listened to masking noise at 65 dB SPL. At 2 s, target sentence embedded in the same level of noise kicks in. At the sentence offset, the masking noise continues for another 2 s to wait for the pupil peak dilation to emerge. All sample points are baseline corrected and normalized following the equation in data pre-processing section. Panel **(B)** shows PPD at 0 dB, 4 dB, 8 dB, 12 dB, 16 dB, and 20 dB SNR levels. Each colored dot corresponds to the average performance of each participant. Black dot corresponds to the average performance across 17 participants, and the error bar corresponds to 1 SE from the mean.

Figure 2 shows the aggregated pupil traces (Figure 2A) and PPD across SNR levels (Figure 2B).

A significant effect of SNR condition on PPD was found (*χ*^2^ = 13.23, df = 5, p = 0.02). Post-hoc z-tests showed significant comparisons as: SNR4 > SNR0 (β = 0.022, SE = 0.008, *p* = 0.007), SNR16 > SNR0 (β = 0.028, SE = 0.008, *p* < 0.001).

Polynomial shape analysis showed no significant linear trend in PPD from SNR0 to SNR20 (*χ*^2^ = 0.89, df = 1, *p* = 0.35, β = 0.04, SE = 0.04, *p* = 0.34), and no significant quadratic trend (*χ*^2^ = 3.82, df = 1, *p* = 0.051, β = −0.06, SE = 0.03, *p* = 0.056), suggesting that at a group level there was no consistent increasing/falling or U-shape/inverse U-shape.

### 3.3 Individual variances in predicting the psychometric curve

**Figure 3:**
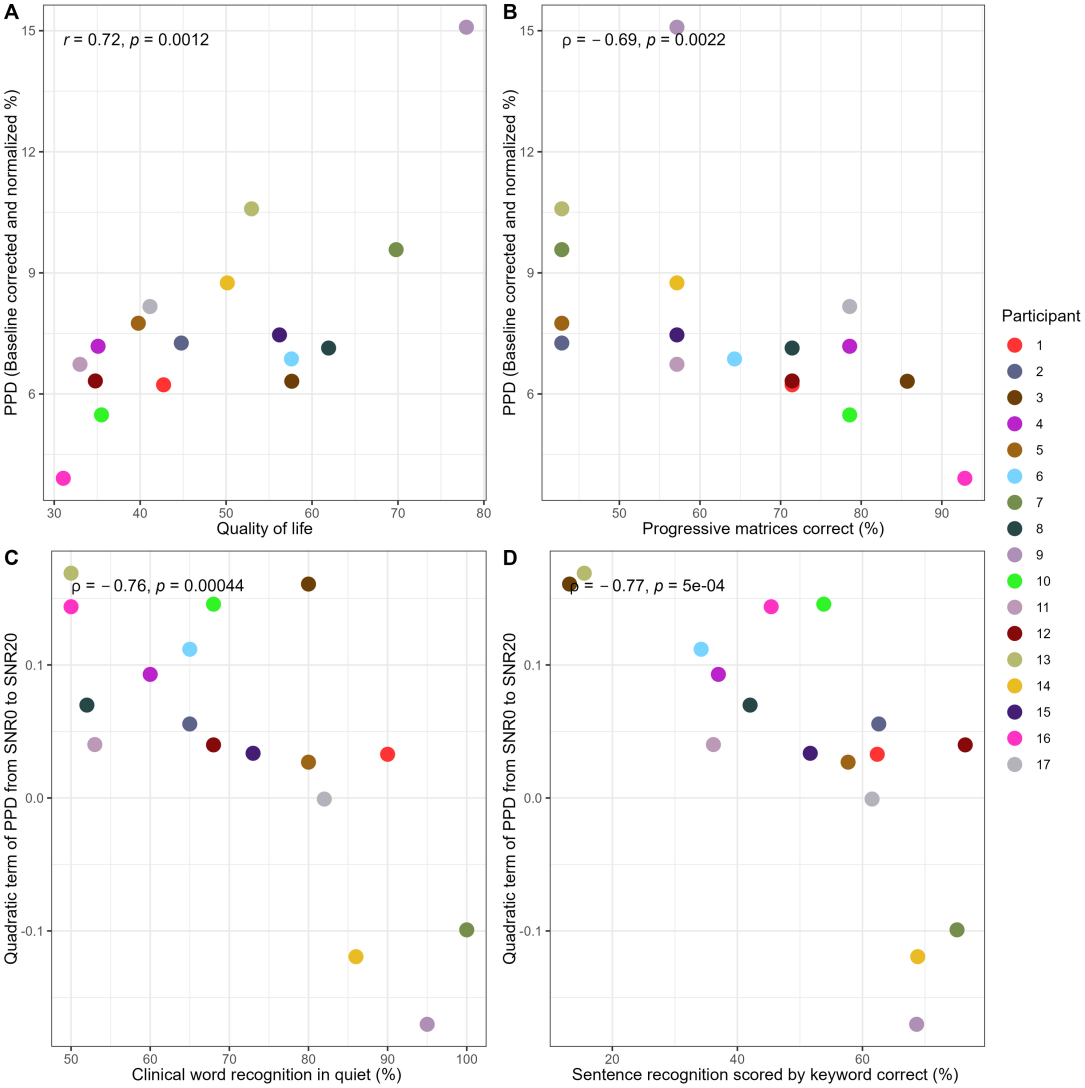
Plots of significant correlation between psychometric shape parameters and individual variabilities. Colored dots correspond to each participant. Panel **(A)** shows the scatterplot between QoL and PPD. Panel **(B)** shows the scatterplot between progressive matrices score and PPD. Panel **(C)** shows the relation between clinical word recognition in quiet and the quadratic term of PPD from SNR0 to SNR20. Panel **(D)** shows the scatterplot between sentence recognition in noise and the quadratic term of PPD from SNR0 to SNR20. Statistical results are presented on the top left of each panel.

Table 2 shows results of correlation tests between individual factors and performances in speech in noise recognition task. Figure 3 shows plots of significant correlations, after Bonferroni correction. For complete distribution and correlation visualizations for all individual factors, see Supplementary Material 2.

**Table 2:** Table with correlation test results.

	Age	CI dur	Word Rec	*T*-Level	QoL	NASA-TLX	Sentence correct in noise	Matrices correct	Stroop correct interference	Stroop control reaction time	Nback dprime	SMRT threshold
PPD	*t* = −1.17, *r* = −0.29, *p* = 0.26	*t* = −1.22, *r* = −0.3, *p* = 0.24	S = 530.3, rho = 0.35, *p* = 0.17	*t* = 0.94, *r* = 0.24, *p* = 0.36	***t* = 3.99, *r* = 0.72, *p* = 0.001**	S = 1,050, rho = −0.54, *p* = 0.03	S = 592, rho = 0.27, *p* = 0.29	**S = 1378.7, rho = −0.69, *p* = 0.002**	S = 520.18, rho = 0.36, *p* = 0.15	*t* = 1.22, *r* = 0.30, *p* = 0.24	*t* = −0.37, *r* = −0.95, *p* = 0.72	*t* = 1.72, *r* = 0.41, *p* = 0.11
Quadratic term of PPD psychometric curve	*t* = 2.04, *r* = 0.47, *p* = 0.06	*t* = 1.81, *r* = 0.42, *p* = 0.09	**S = 1433.5, rho = −0.76, *p* = 0.0004**	*t* = −0.53, *r* = −0.13, *p* = 0.61	*t* = −2.10, *r* = −0.48, *p* = 0.05	S = 308, rho = 0.55, *p* = 0.03	**S = 1,442, rho = −0.77, *p* = 0.0003**	S = 502.58, rho = 0.38, *p* = 0.13	S = 858.26, rho = −0.05, *p* = 0.84	*t* = 0.02, *r* = 0.006, *p* = 0.98	*t* = 0.47, *r* = 0.12, *p* = 0.64	*t* = −3.09, *r* = −0.62, *p* = 0.007

Each column contains shape parameters relating to the psychometric relation between task difficulty (SNR) and pupillary response (PPD). Each row contains individual performance and top-down/bottom-up variabilities. Results in bold are correlation tests that remain significant after Bonferroni correction (*p* < 0.002).

**Figure 4:**
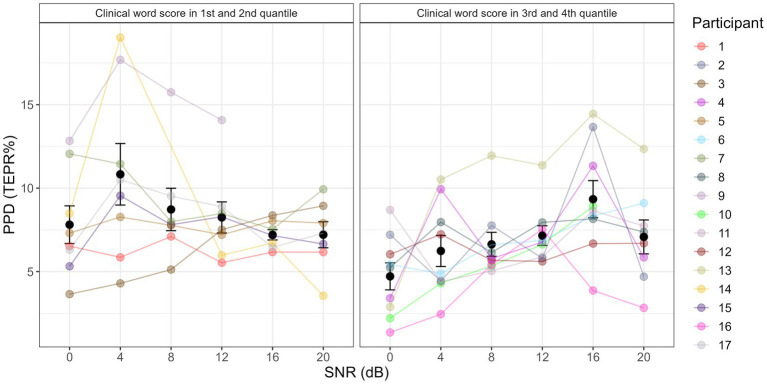
PPD results at different SNR levels, with participants split into two groups. Left panel shows results for participants whose clinical word scores are in the 1st and 2nd quantile, and 4 dB SNR condition was significantly higher than 0 dB, 16 dB, and 20 dB SNR. The right panel shows results for participants whose clinical word scores are in the 3rd and 4th quantile, and 16 dB SNR condition was significantly higher than 0 dB, 4 dB, 8 dB, 12 dB, and 20 dB SNR.

To further interpret the consistent change and significant correlation between clinical word recognition and the progression of the quadratic term of PPD from SNR0 to SNR20 (Figure 3C, rho = −0.76, *p* = 0.0004), 17 participants were split into two groups from the median based on their clinical word recognition performance. Same data used to plot Figure 2B were used to plot the results of two sub-groups in Figure 4. Same analysis methods used for investigating the first hypothesis (i.e., whether a consistent psychometric relation exist) was performed on two groups, to validate visual observations in Figure 4. Accordingly, this additional fixed effect factor termed wordgroup with two levels (1st, 2nd quantile and 3rd, 4th quantile of word recognition) was entered into the mixed effect models. Same model and post-hoc testing methods were performed to examine the main effect of wordgroup and its interaction with SNR condition on PPD and polynomial shapes. Note that this sub-group analysis intends to visualize and support the interpretation of a significant across-participant correlation that is already reported within the scope of our second hypothesis (i.e., significant correlation between PPD psychometric curve quadratic term and word recognition (rho = −0.76, p = 0.0004)). Therefore, this extra investigation does not impair the statistical integrity of the main analysis by increasing the chances of false positives. Splitting from the median of the clinical word recognition would not bias our interpretation, considering that there was a consistent across-participant correlation. (For sanity check on this point, see Supplementary Material 3 where CI participants are split into 1st and 4th quantile. With fewer participants and more different word recognition performance in each sub-group, results remain similar to using median split.) Independent sample t-tests verified that the two sub-groups did not differ in other profiles such as age (*t* = −1.53, df = 10.51, *p* = 0.16), mean T-level (*t* = 1.45, df = 14.20, *p* = 0.17), CI duration (*t* = −1.31, df = 12.27, *p* = 0.21), QoL (*t* = 1.82, df = 13.84, *p* = 0.09), SMRT threshold (*t* = 1.81, df = 14.73, p = 0.09), Nback d-prime (*t* = 0.73, df = 13.58, *p* = 0.48), Stroop interference score (*t* = 0.8, df = 9.23, *p* = 0.44), nor speech in noise task performance (*t* = 1.40, df = 14, *p* = 0.18).

There was no significant main effect of wordgroup on PPD (*χ*^2^ = 2.69, df = 1, *p* = 0.10), suggesting that the two subgroups did not differ in average PPD. There was a significant interaction between SNR and wordgroup on PPD (*χ*^2^ = 13.72, df = 5, *p* = 0.02). In the group where participants were in the 1st and 2nd quantile of clinical word recognition, SNR4 > SNR0 (β = 0.03, SE = 0.01, *p* = 0.01), SNR16 > SNR4 (β = −0.03, SE = 0.01, *p* = 0.03), SNR20 > SNR4 (β = −0.02, SE = 0.01, *p* = 0.04). In the group where participants were in the 3rd and 4th quantile of clinical word recognition, SNR12 > SNR0 (β = 0.02, SE = 0.01, *p* = 0.03), SNR16 > SNR0 (β = 0.05, SE = 0.01, *p* < 0.001), SNR20 > SNR0 (β = 0.02, SE = 0.01, p = 0.04), SNR16 > SNR4 (β = 0.03, SE = 0.01, *p* = 0.005), SNR16 > SNR8 (β = 0.03, SE = 0.01, *p* = 0.008), SNR16 > SNR12 (β = 0.02, SE = 0.01, *p* = 0.02), SNR20 < SNR16 (β = −0.025, SE = 0.01, *p* = 0.01). All the post-hoc tests were consistent with the pattern in Figure 4.

There was a significant interaction between wordgroup and the linear shape (*χ*^2^ = 6.03, df = 1, *p* = 0.01), suggesting that high word recognition group had more falling trend than low word group progressing from SNR0 to SNR20 (β = 0.18, df = 11.67, *p* = 0.03).

## 4 Discussion

In this study, with CI users we revisited the well-established psychometric relation between speech recognition and pupillary response reported in NH and HI listeners (Ohlenforst et al., 2017, 2018; Wendt et al., 2017). By replicating the experiment on a group of post-lingually deaf CI users, we constructed a similar psychometric function across a wide range of SNR and intelligibility levels. We also measured individual variabilities in audiological status, auditory sensitivity, and cognitive profile, to examine their strength in predicting individual psychometric curves.

### 4.1 PPD relates to listening effort (in the right region of the PPD psychometric curve)

**Figure 5:**
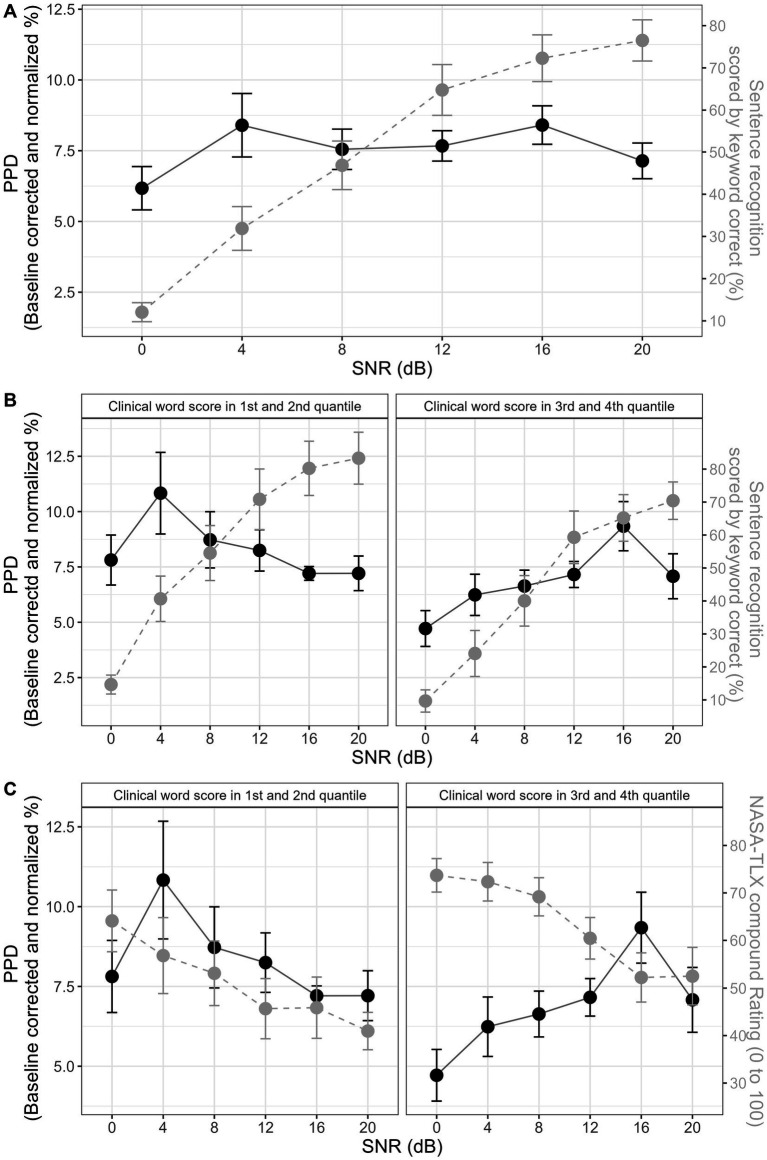
Double psychometric function figures for PPD, sentence recognition in noise and NASA-TLX. In all panels, dot corresponds to the average performance, and the error bar corresponds to 1 standard error (SE) from the mean. Panel **(A)** combines dots and error bars in black solid line to indicate PPD psychometric function, and gray dashed line to indicate sentence recognition psychometric function. Panel **(B)** splits participants into two group, with the left side containing participants whose clinical word recognition falls in the 1st and 2nd quantile and the right-side 3rd and 4th quantile. Gray dashed lines in panel **(C)** connect dots and error bars to indicate NASA-TLX psychometric functions.

At a group level, CI results showed no consistent inverse U-shape as in NH and HI listeners. To aid direct comparison with previous studies, Figure 5A joins the information in Figure 1A and Figure 2B to re-plot the same double psychometric function figure as in previous studies (Ohlenforst et al., 2017, 2018; Wendt et al., 2017). Figure 5A shows no consistent increasing/decreasing trend or U-shape/inverse U-shape in PPD, from 0 dB to 20 dB SNR, which covers a wide range of difficulty and intelligibility. Note that failure to find the same relation is not due to bias in participants selection or errors in experimental design. CI users in this study show comparable audiological profile, speech-in-noise, and clinical word recognition performance as the general CI population (Holden et al., 2013; Goudey et al., 2021). Pupil traces and obtained PPD are also comparable with past studies (Winn et al., 2015; Wagner et al., 2019; Russo et al., 2020). Furthermore, consistent subjective effort ratings indicate that participants experience distinctive levels of effort across different conditions. Therefore, our experimental manipulation has succeeded in varying intelligibility and perceived listening effort, yet the effect is not shown in PPD.

Anecdotally, there have been reports from clinical and research sites that pupillometry has not been reliably reflecting changes in experimental manipulations for CI users, casting doubts on the suitability of pupillometry as a tool for CI applications. And our results seem to agree with the anecdotal reports from the field. This is worrying, because if pupillary response does not show any consistent variation pattern across various levels of perceived listening effort in CI users, then pupillometry cannot be used as a tool to reliably assess listening effort and inform research and clinical practice.

However, when the great individual variability in clinical word performance is considered, results show a significant correlation between psychometric curve and clinical word performance (rho = −0.76, *p* = 0.0004). Similarly, to aid interpretation and direct comparison with previous studies, Figure 5B joins the information in Figures 1A, 3 to re-plot the double psychometric curves. Again, as emphasized in the analysis, although Figures 4, 5B arbitrarily split CI participants into two groups based on the median clinical word recognition, the significant inter-participant correlation indicates that the pattern shown in the two figures is consistent from the lowest to the highest CI performers. A significant correlation between sentence recognition in noise and psychometric curve further supports this pattern (rho = −0.77, *p* = 0.0003). This suggests that pupillometry data in CI users are not entirely chaotic but driven by individual differences in speech intelligibility. As shown in Figure 5B, CI users display a similar inverse U-shape in PPD psychometric curve as NH and HI listeners but differ in the location of the “tipping point” of the curves. In the lower quantile group (i.e., low word recognition), the “tipping point” occurs at 16 dB SNR or around 40% intelligibility, and in the higher quantile group (i.e., high word recognition), the “tipping point” occurs at 4 dB SNR or around 70% intelligibility. Not surprisingly, when combining the two groups into one figure (Figure 2B), the mean psychometric curve appears flat.

Furthermore, speech intelligibility may not be the only difference between NH/HI listeners and CI users. It is no news that the same SNR level does not yield same speech recognition scores among NH, HI, and CI listeners, so comparing directly ‘tipping points’ in terms of their corresponding SNR levels is not thorough enough. Regardless of the difference in SNR levels, NH and HI listeners typically showed the ‘tipping point’ on their pupillometry psychometric curves at around 40–50% speech intelligibility (Ohlenforst et al., 2017; Wendt et al., 2017; Dingemanse and Goedegebure, 2022). This suggests that even though NH and HI listeners have different speech recognition, they tend to disengage from the task at similar intelligibility level. But CI users show great difference in the intelligibility level where the ‘tipping point’ occurs (Figure 5B). It seems that depending on which quantile of clinical word recognition performance CI users land in, they disengage from the task at either high (~70%) or low intelligibility level (~40%) in speech-in-noise tasks. For instance, for CI users in lower quantile group (Figure 5B left panel), even at testing levels when sentence recognition is successful (say, >60%), PPD increases with increasing intelligibility or easier SNR. At the same speech intelligibility region, CI users in higher quantile group show the opposite trend, where PPD decreases with increasing intelligibility or easier SNR. Could it be that CI users also differ in subjective decision (consciously or unconsciously) of when to disengage from a task? This mechanism could explain the difference in the consistency of the “tipping point” observed in NH/HI listeners and in our CI users. For instance, even though relatively low-performing CI users can perform successful speech recognition, the cost of such an effort is deemed too huge to be rewarding or motivating, hence translating into an “inversion” in physiological markers and negative listening effort that could further evolve to listening fatigue. In our current study, NASA-TLX is a subjective measure of effort that does not include the dimension of motivation and engagement. At a group level, NASA-TLX also related to pupillometry psychometric curve, but did not pass Bonferroni correction (rho = −0.54, *p* = 0.03 for correlation with the average PPD; rho = 0.55, *p* = 0.03 for correlation with the quadratic term). A similar combination of Figures 1B, 3B shows that, interestingly, both ‘tipping points’ in the two groups occur at around 50% NASA-TLX score, a similar value at which NH/HI listeners disengage but in terms of objective intelligibility (Figure 5C). According to the Framework for Understanding Effortful Listening (FUEL), the allocation of limited cognitive resources for the listening task is influenced by the listener’s motivation (Pichora-Fuller et al., 2016). Effort investment will increase with task demands, as long as there is motivation to succeed at the task and the goal is deemed attainable (Brehm and Self, 1989). Maximal effort is the point when the listener is sufficiently motivated to succeed in communication, and listening is demanding but not perceived as wasted effort (Richter, 2013, 2016; Pichora-Fuller et al., 2016; Herrmann and Johnsrude, 2020). It is likely that the ‘tipping point’ observed in PPD psychometric function could be the physiological biomarker of such maximal effort, where further task demands will result in de-motivation and task being deemed as not worthwhile. Past pupillometry studies have shown pupil dilation is sensitive to different motivation levels and conditions, due to its connection with the locus coeruleus noradrenergic system that is responsible for regulating task engagement (Murphy et al., 2011; Carolan et al., 2022). And we further hypothesize that pupillary response can be sensitive enough to reflect the threshold where task engagement starts to decrease. Note that the interpretation of the ‘tipping point’ as point of disengagement or ‘giving up’ has not been validated in past studies, for instance using a subjective questionnaire on motivation or engagement. Our current study does not have the correct subjective questionnaire for quantifying motivation or engagement, nor enough power to examine this interpretation statistically, due to a lack of a measure of motivation or engagement and the fear of inflated false positive rates. Therefore, future studies need to incorporate measures of motivation or engagement to uncover the ecological meaning of the ‘tipping point’ and validate its sensitivity and reliability as a measure of disengagement in clinical populations.

### 4.2 Individual factors affect individual PPD psychometric function

Despite the big individual differences in pupil psychometric curves, individual differences measured in life status and neurocognitive factors explain certain amount of variability significantly (mean *R*^2^ = 0.54, *p* < 0.002 after Bonferroni correction). Non-verbal intelligence measured by Progressive Matrices is negatively correlated with the average PPD, suggesting that CI participants with higher non-verbal intelligence showed lower PPD across all SNR conditions. The relation between non-verbal intelligence and speech outcomes in CI users have been examined in past studies, but it was unclear how non-verbal intelligence affect listening effort (Mattingly et al., 2018; Moberly et al., 2019; Amini et al., 2023; Beckers et al., 2023). Non-verbal intelligence is typically found to relate positively with word or sentence recognition, indicating that higher ability to induce abstract relation helps recognizing sentences that have degraded resolution due to CI signal processing (Carpenter et al., 1990). Similarly, better non-verbal intelligence should also help relieve listening effort by listeners having larger cognitive capacities at disposal. However, no significant correlation was found between non-verbal intelligence and quadratic terms of PPD psychometric curve (unlike clinical word and sentence-in-noise recognition), suggesting that non-verbal intelligence is not associated with the psychometric function curvature. It is possible that individual cognitive capacities can modulate the relation between listening effort and performance for a given task, but not necessarily the structural relation with the point of de-motivation or dis-engagement when facing various levels of difficulties.

QoL measured by NCIQ related positively with the average PPD, suggesting that CI participants with higher quality of life ratings showed higher PPD across all SNR conditions. This result seems to be counter intuitive and inconsistent with past findings (especially when there is a positive correlation between QoL and word recognition performance, see Supplementary Material 2). For instance, past studies have reported that higher Speech, Spatial and Qualities (SSQ) related with smaller pupillary response in CI users at 10 dB SNR and quiet conditions (Russo et al., 2020); higher subjective daily fatigue related with bigger PPD (Wang et al., 2018). Arguably, NCIQ contains more domains than subjective hearing status and daily fatigue, and includes measures on physical (basic sound perception, advanced sound perception, speech production), psychosocial (self-esteem) and social (activity limitations, social interactions) functioning, hence could be affected by various cognitive, sensory, and demographic factors (Skidmore et al., 2020). It is still unexpected that the direction of correlation is the opposite. One possibility could be that the average PPD, as a psychometric function shape parameter in our current study, reflects more than explicit cognitive resources allocated to the task, but also the multidimensionality of listening effort (Alhanbali et al., 2019; Francis and Love, 2020; Shields et al., 2023). Therefore, future studies with bigger sample size should explore and add mediating factors (for instance, level of engagement, likelihood of giving up, socio-economic status) to disentangle the multidimensionality of this physiological measure. QoL also related to the quadratic terms of individual PPD psychometric curves in the same direction as clinical word recognition (i.e., participants with higher clinical word and higher QoL had similar PPD psychometric curves than their counterparts) but did not pass Bonferroni correction. It seems that QoL might affect individual variabilities when listeners disengage or perceive the task as unworthy of effort investment. An individual with better status of life quality might maintain motivation or engagement during communication, even when the auditory scene is challenging. Factors that could affect individual decision to disengage need to be explored in future studies.

**Figure 6:**
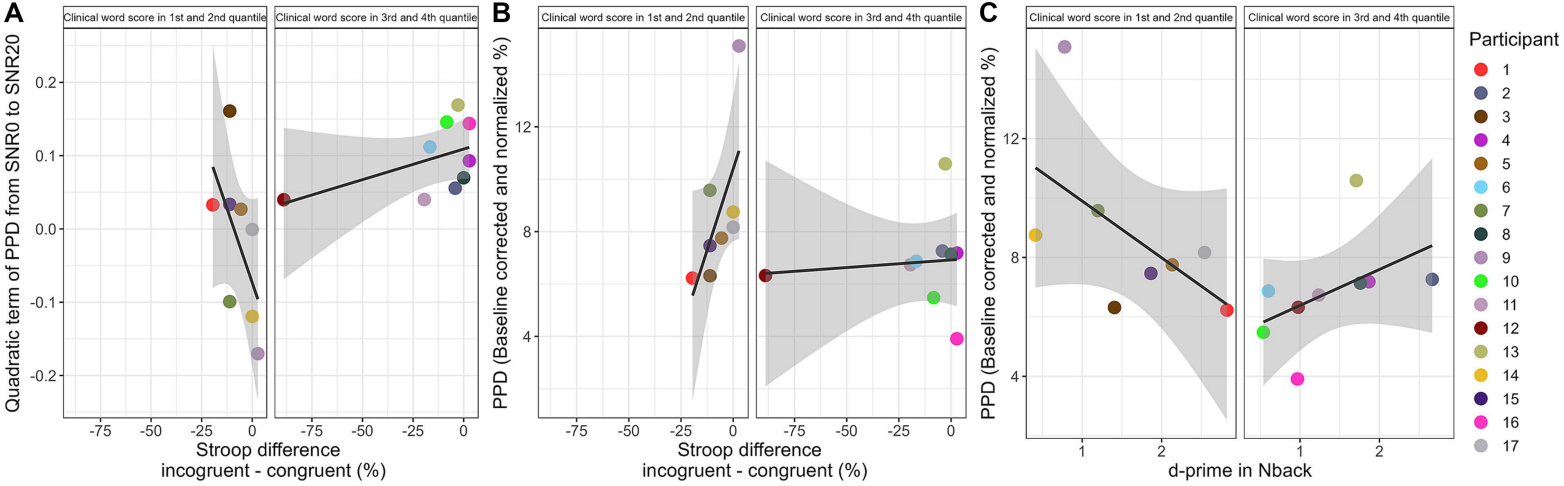
The relationship between individual Stroop difference score and the quadratic term of PPD from SNR0 to SNR20 Panel **(A)**, and the PPD Panel **(B)** are shown for the two groups. Panel **(C)** shows the relationship between d-prime score and the PPD..

Some absences of significant relations are surprising. Spectro-temporal auditory acuity has been found to relate to speech recognition in noise and sensitive enough to reliably discriminate CI users (Tamati et al., 2020; Moberly et al., 2021). Therefore, it should not be difficult to assume that this bottom-up factor can also explain individual differences in listening effort and PPD psychometric functions. However, SMRT performance related to the quadratic term of individual PPD psychometric curves in our current study but did not pass the Bonferroni correction. Top-down factors measured in our study relating to working memory and inhibition also did not significantly explain variabilities in average PPD or psychometric functions, despite reports on the possible importance of these cognitive domains on CI users’ speech recognition. One explanation to the lack of findings could be due to the mixed results in past studies and variabilities in tests used to measure these cognitive domains and speech recognition performance (Tao et al., 2014; Skidmore et al., 2020; Tamati et al., 2020; Völter et al., 2021, 2022; Beckers et al., 2023). For instance, different tests have been used to measure working memory, and results in significance level and effect size differ, also depending on which speech recognition outcomes the cognitive tests are related to. Indeed, an increasing number of studies in recent years have started to investigate the impact of cognitive factors on the variability of CI outcomes, but we are still far away from producing a consistent synergy of knowledge. There is still no consensus on the effect size of the relation and what the best tests to quantify these cognitive domains are. And if unexplained variability in CI speech performance is still an issue, the large unexplained variability in CI listening effort that we have observed is not surprising. Another possibility for the lack of significant findings could be that the importance of top-down and bottom-up factors to CI hearing outcomes depends on whether CI user lands in high-or low-performing group. For instance, CI users with high performance using Perpetually Robust English Sentence Test Open-set (PRESTO) also had better SMRT and non-verbal intelligence, but CI users with low PRESTO had more variable individual profiles (Tamati et al., 2020). CI users with low monosyllabic word recognition (<30%) tend to have significant differences in attention and working memory, while CI users with high word recognition (>70%) tend to take advantage of top-down benefits more consistently (Völter et al., 2021). It seems that bottom-up auditory sensitivities are important for CI users to accumulate enough information, but once a certain amount is reached, then the top-down neurocognitive capacity contributes to extra benefit in the hearing outcomes. Therefore, it is likely to have better cognitive capacities that could specifically benefit high performers. In our current study, CI participants were split by word recognition in the median, hence the range of performance was limited (low word group: 50 to 68%; high word group: 73 to 100%). Participants agreeing to attend experiments were already comfortable with doing speech in noise task, a demanding task for CI users, so arguably, our participants were relatively good performers in our CI participants’ pool. Therefore, our experiment design is not suitable for comparing peripheral and neurocognitive interactions in bottom-and top-performers. However, our results suggested a similar trend. From low to high word recognition performers, there was a consistent change in PPD psychometric function and SMRT was associated with that consistent change (*r* = −0.62, *p* = 0.007, did not pass Bonferroni correction). When examining whether there is also a group difference in the relation between PPD psychometric function and neurocognitive factors as suggested by past literature, we discovered that it was indeed the case for Stroop and N-back results (Figure 6). It seems that the association between these neurocognitive factors and pupil psychometric function, if exists, might only be exhibited in the high word recognition group. For participants with high word recognition, higher difference between congruent and incongruent performance was associated with smaller quadratic term (β = −0.81, SE = 0.36, p = 0.04), and no significant relationship for participants with low word recognition (β = 0.08, SE = 0.08, *p* = 0.36); for participants with high word recognition, higher difference between congruent and incongruent performance was associated with bigger average PPD (β = 0.25, SE = 0.1, *p* = 0.03), and no significant relationship for participants with low word recognition (β = 0.005, SE = 0.03, *p* = 0.82); For participants with high word recognition, higher d-prime sensitivity was associated with smaller average PPD (β = −0.01, SE = 0.01, *p* = 0.06), more so compared to participants with low word recognition (β = −0.07, SE = 0.02, *p* = 0.02). As a cautious note, these interactions are observed post-hoc, without the explicit control on participants group, and fail strict Bonferroni correction on value of ps. Therefore, future studies should improve on these points to confirm the relationship.

### 4.3 Limitations

Although our current study sets out with explicit hypothesis on the pupillometry psychometric shape in CI users and its relationship with auditory and neurocognitive factors, there are many interesting post-hoc findings that can be examined and explored. These include, for instance, replicating past findings in the construct relationship across cognitive tests, exploring not main but mediating effects of hearing status and individual differences on hearing outcomes, replicating construct validities of listening effort measures etc. Some of these results are not included in the main paper but can be found in Supplementary Material 2, reported in a descriptive manner. We also did not explore all pupillometry indices, because the aim of the current experiment was to examine whether PPD, the most widely reported listening effort index, can consistently reflect listening effort as in NH and HI listeners. Supplementary Material 2 also provides examination on other pupillary responses (i.e., pupil peak latency and pupil baseline). Future studies with bigger sample size, hence with stronger statistical power, should focus on exploring and validating those relations. Note that past studies have pointed to pupil baseline as a biomarker for variations in arousal and engagement, which could be responsible for the individual differences in pupil psychometric function and the ‘tipping point’ (Ayasse and Wingfield, 2020; Alhanbali et al., 2021; Micula et al., 2022). Unfortunately, the eye tracker used in our experiment did not measure (in hardware) pupil diameter in absolute unit mm, making the comparison across recording sessions inaccurate. Also, CI participants were prioritized to receive breaks whenever needed, probably affecting arousal and engagement levels independently from the experimental design.

While it is important to replicate past studies and explore new relationships, it is also important to control for family-wise error rate, especially in the context of exploring and discovering new and impactful factors to explain and predict CI hearing outcomes. In fear of type 1 error, i.e., rejecting a null hypothesis that is true in the population, we have applied strict multiple testing value of p corrections, even though this practice is not consistently done in the past literature. Ideally, a structural approach can be more suitable to identify and quantify the importance of individual factors by also considering other factors in the structure. But supporting such models requires bigger dataset and more measurements of different cognitive, listening effort, hearing status and audiology domains (Marsja et al., 2022; Homman et al., 2023).

### 4.4 Practical considerations in research and clinical applications

This study is motivated by the authors intending to resolve anecdotal reports on using pupillometry with CI users in the clinical settings. Past studies have examined CI users’ pupillary response at various points on the psychometric curve (Russo et al., 2020; Stronks et al., 2021; Dingemanse and Goedegebure, 2022), however our study is the first to date to approximate a more complete curve by testing multiple points and considering a wide range of individual differences. The results and their implications need to be carefully digested for research and clinical application.

Firstly, pupillometry remains a reliable method to quantify listening effort, but specifically in a region where speech communication is successful or deemed worthwhile. This is not a foreign concept, considering the guidelines for measuring listening effort in NH and HI listeners (Winn et al., 2018; Keidser et al., 2020). But it is more difficult to implement in CI users, due to their big variabilities in speech outcomes and motivation, as our results have shown. For instance, the assumption that 50% intelligibility is where CI users show PPD peak on their psychometric function might not stand for every CI user. For some CI users, PPD is associated with listening effort and perceived task demand when speech intelligibility is above 60–70%. Apart from careful selection of testing levels, it is still unclear whether and how much engagement or motivation affects PPD psychometric function. However, judging by the difference in the variability of psychometric curves in CI users compared to NH and HI listeners, engagement or motivation probably plays a bigger role in CI users. Therefore, using more ecological speech materials that reflect real communication demands can simulate better the real motivation of CI users to perform successful daily communication (for instance, using well-controlled ecological virtual reality setup as in Pedersen et al. (2023). Arguably, this approach could stabilize the relation between cognitive capacity and effort, and also reveal listening effort in scenarios where it is most relevant (Lunner et al., 2016; Winn et al., 2018; Herrmann and Johnsrude, 2020). Future work on seeking optimal conditions to capture CI users’ listening effort in research labs and clinics should consider variabilities in their performance level and motivation.

Secondly, individual differences in CI users are not trivial. Our results join the past literature in showing that top-down and bottom-up individual differences affect CI performance outcomes, but also adds in further complexities by including the dimension of listening effort (Amini et al., 2023; Beckers et al., 2023). Our result also highlights not just the importance of investigating the construct validity across listening effort measures, but also the link between listening effort and QoL. Relationship between objective outcome measures and Quality of Life might not be straightforward, as shown in our results, so mediating factors need to be taken into consideration to fully understand how to use objective measures such as pupillometry to inform and advise patients’ care. It could be beneficial to be aware of the importance of individual variabilities in hearing outcomes at direct points of care (hospitals, audiology clinics, rehabilitation centers and research labs), so that a holistic and responsible database can be built that contains individual factors such as hearing status, neurocognitive functioning, auditory sensitivity, socio-economic status, surgical outcomes etc. (see^2^ for an example). Such database should also follow strict privacy and ethics compliance such as General Data Protection Regulation (GDPR). Further guidelines could be helpful to be agreed upon across multiple sites on what are the criteria for the discovery of meaningful individual factors, such as what are acceptable type 1 and type 2 errors rates, what are clinically relevant effect sizes, how to include mediating factors, how to discover bias in database and site experimental practices, etc.

## 5 Conclusion

To summarize, our study replicates, in CI users, the well-validated NH and HI listeners’ inverse U-shape psychometric function between pupillary response and speech intelligibility across a wide range of SNR levels. This psychometric relationship supports our interpretation of pupillary response as a biomarker of listening effort, hence important for both research and clinical practices. Our results suggest that big variabilities in individual psychometric relationships disturb the construction of a consistent psychometric function at the group levl. However, some individual factors relating to CI users’ speech outcomes, quality of life and neurocognitive functioning can predict individual psychometric shapes. Future studies should examine the possible effect of motivation or engagement in modulating CI users’ occurrence of ‘tipping point’ on their psychometric functions. It is likely that a new biomarker indicating when CI users ‘give up’ could be an important holistic benchmark for future CI strategy and rehabilitation innovations to validate their effects in improving CI users’ engagement in speech communication.

## Data availability statement

The raw data supporting the conclusions of this article will be made available by the authors, without undue reservation.

## Ethics statement

The studies involving humans were approved by Ethics Committee of the Hospital Universitario Virgen Macarena in Seville, Spain (PEIBA#19/2019). The studies were conducted in accordance with the local legislation and institutional requirements. The participants provided their written informed consent to participate in this study.

## Author contributions

YZ: Conceptualization, Data curation, Formal analysis, Investigation, Methodology, Project administration, Software, Supervision, Validation, Visualization, Writing – original draft, Writing – review & editing. MC-L: Conceptualization, Data curation, Formal analysis, Investigation, Methodology, Project administration, Resources, Supervision, Validation, Writing – original draft, Writing – review & editing. AP-R: Data curation, Investigation, Project administration, Validation, Writing – review & editing. SB-T: Formal analysis, Validation, Visualization, Writing – review & editing. FP: Conceptualization, Funding acquisition, Investigation, Methodology, Project administration, Resources, Supervision, Validation, Writing – review & editing. SS-G: Funding acquisition, Project administration, Resources, Supervision, Writing – review & editing.

## Footnotes

^1^https://docs.pupil-labs.com/

^2^https://plataforma.innovacionsalud.org
