# Supplementary material for: Impact of SNR, peripheral auditory sensitivity, and central cognitive profile on the psychometric relation between pupillary response and speech performance in CI users
Source: Front Neurosci. 2023 Dec 21;17:1307777. doi: 10.3389/fnins.2023.1307777 (PMC10768066; doi:10.3389/fnins.2023.1307777)
Supplement: Supplementary file 1 [file Data_Sheet_1.docx]

Supplementary Material 1

Impact of SNR, peripheral auditory sensitivity, and central cognitive profile on the psychometric relation between pupillary response and speech performance in CI users

Yue Zhang^*^, M. Amparo Callejón-Leblic, Ana M. Picazo-Reina, Sergio Blanco-Trejo,

Francois Patou, Serafín Sánchez-Gómez

*** Correspondence:** Corresponding Author: [yuza@oticonmedical.com](mailto:yuza@oticonmedical.com)

This supplementary material includes visualization and analysis of data that are not reported in the main body of the paper, offering a deeper insight into the results obtained. The supplementary material visualizes audiograms obtained using narrow band stimuli, and the between-participant consistency in the measurements obtained.

S1_Fig1 shows the audiogram for each CI user from our cohort. The frequencies employed were 250, 500, 1k, 2k, 3k, 4k, 6k and 8k Hz.


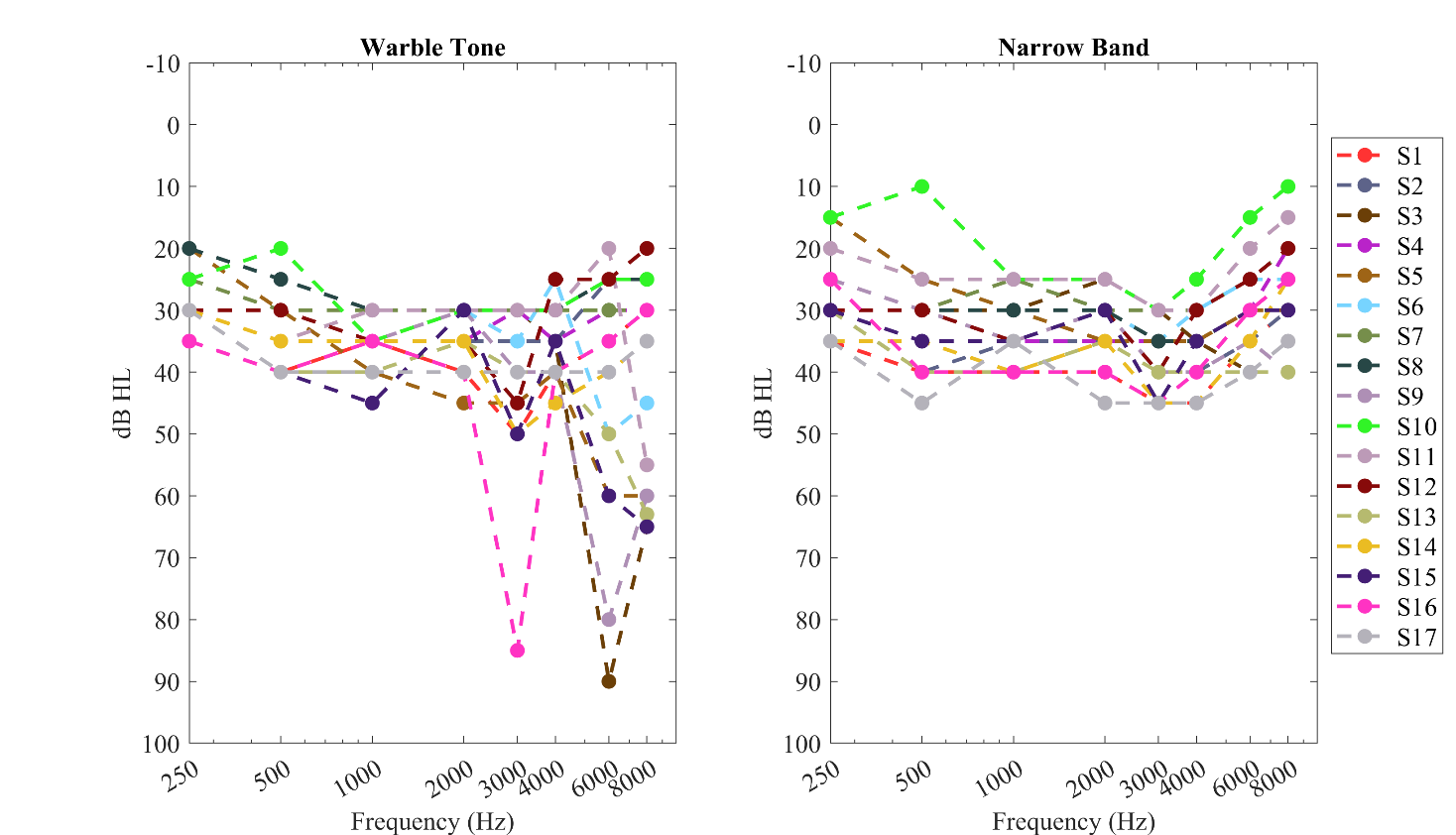


S1_Fig1. Audiograms of all CI participants, using narrow band stimuli.


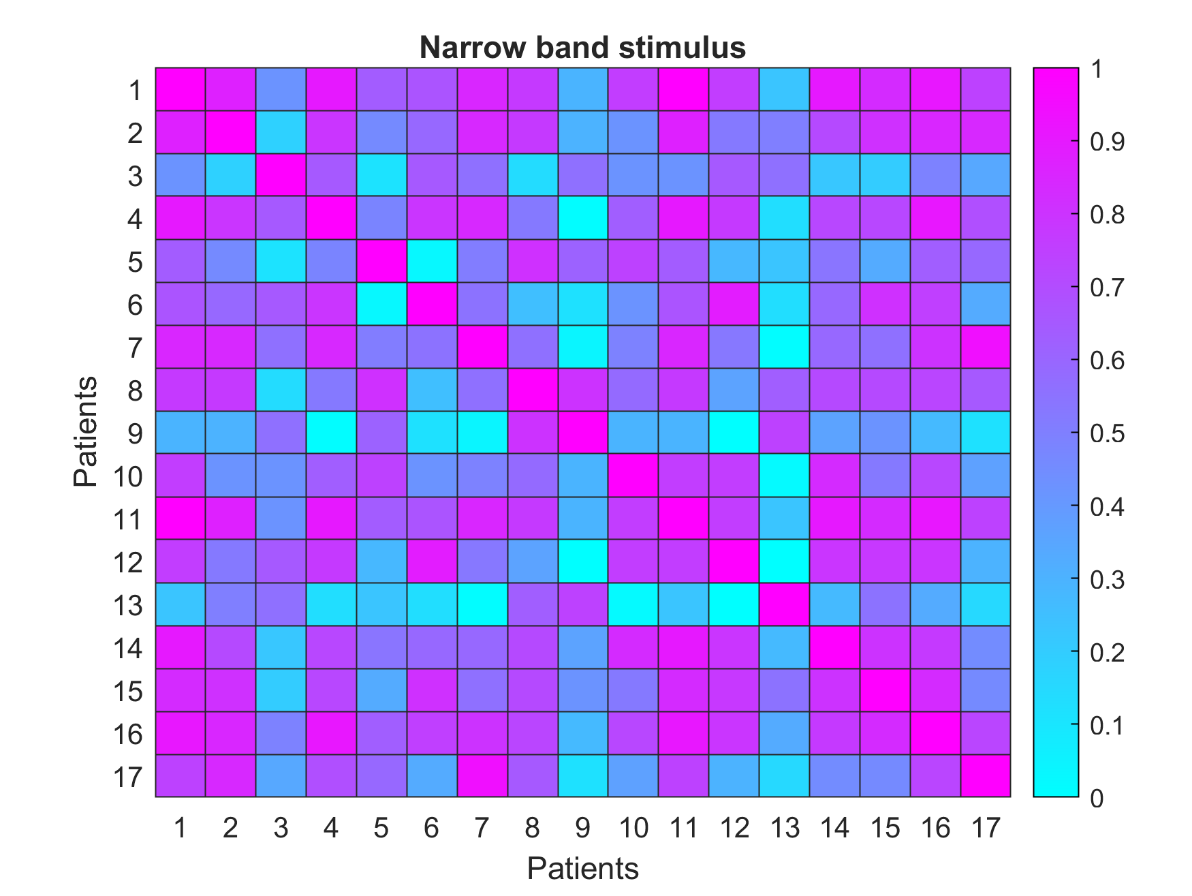


S1_Fig2. Inter-subject correlations of auditory thresholds measured with narrow band stimulus.

**
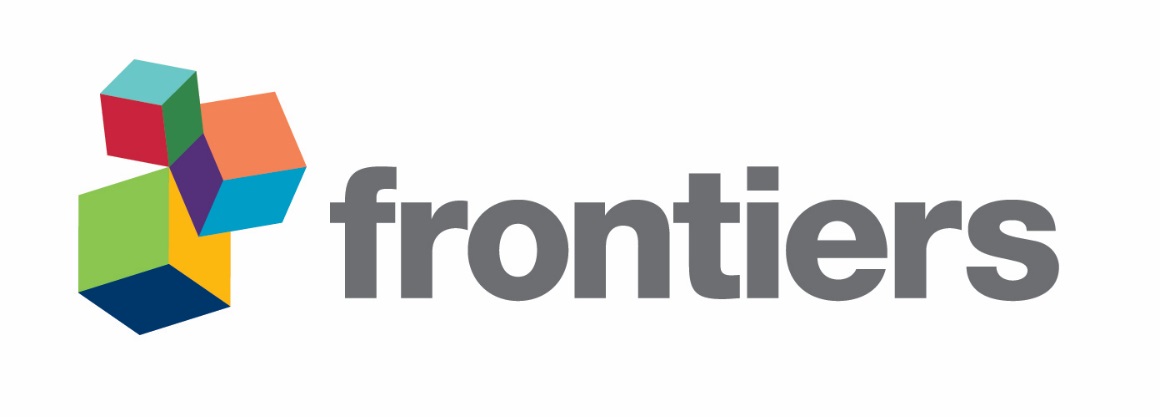
**
