# Supplementary material for: Impact of SNR, peripheral auditory sensitivity, and central cognitive profile on the psychometric relation between pupillary response and speech performance in CI users
Source: Front Neurosci. 2023 Dec 21;17:1307777. doi: 10.3389/fnins.2023.1307777 (PMC10768066; doi:10.3389/fnins.2023.1307777)
Supplement: Supplementary file 2 [file Data_Sheet_2.docx]

Supplementary Material 2

Impact of SNR, peripheral auditory sensitivity, and central cognitive profile on the psychometric relation between pupillary response and speech performance in CI users

Yue Zhang^*^, Amparo Callejón-Leblic, Ana M Picazo-Reina, Sergio B Trejo, Francois Patou, Serafín Sánchez-Gómez

*** Correspondence:** Corresponding Author: yuza@oticonmedical.com

This supplementary material includes visualization and analysis that are not reported in the main body of the paper, to provide more information about CI users’ individual variability and pupillary responses other than PPD. S2_Fig1 shows a correlation matrix among measured CI individual differences in cognitive, auditory, and hearing profile. Note that due to the small sample size (N = 17), the relations examined in S_Fig1 cannot be reliably tested using correlation tests. Therefore, only bivariate distribution, scatter plot with estimated regression fitting and standard error are shown in S_Fig1. Consolidating possible relations in S2_Fig1 statistically requires future studies with bigger sample size.


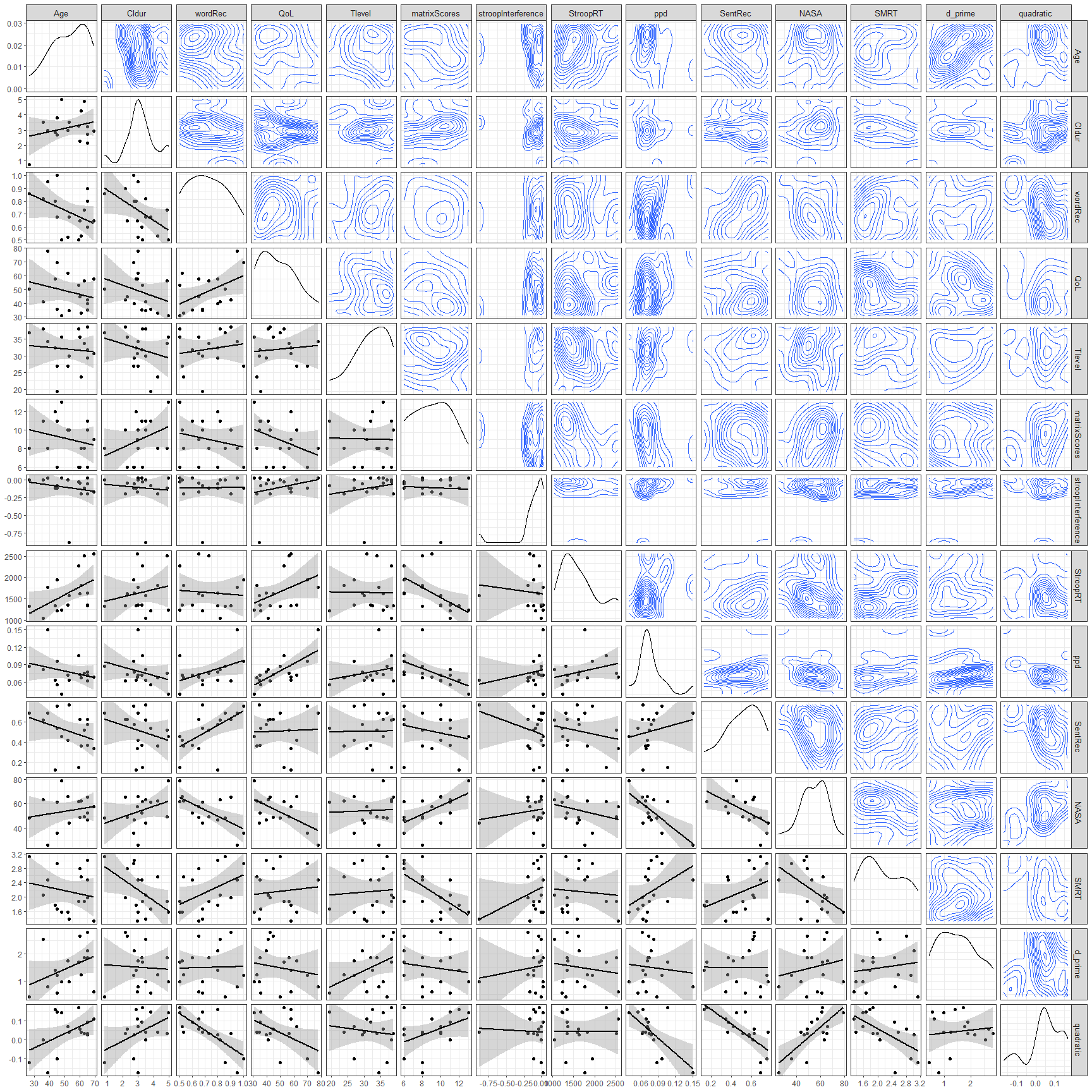


**S_Fig1:** Individual factor correlation matrix. The lower panels show scatterplots and fitted linear regression lines with 1 SE shaded areas. Upper panels show bi-variate distribution. Diagonal panels are the distribution of individual factors.

S2_Fig2 shows the psychometric relation between peak pupil latency and SNR (panel A), and a split of CI participants group using the same rule as Figure 4 (panel B). Peak pupil latency was measured as the time from the onset of the sentence to the time where peak pupil size occurred in the analysis window (from onset of the sentence to the onset of verbal response).

| 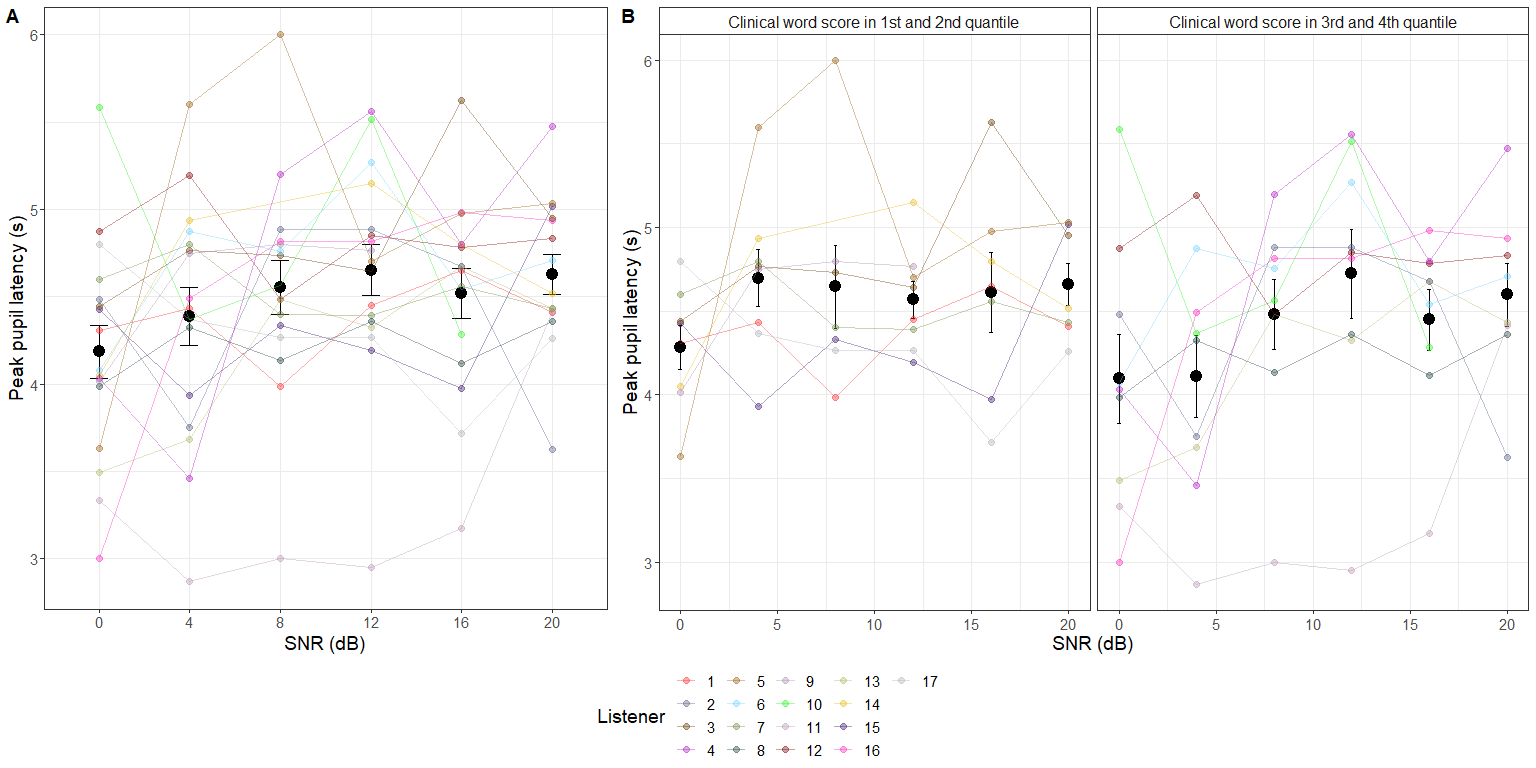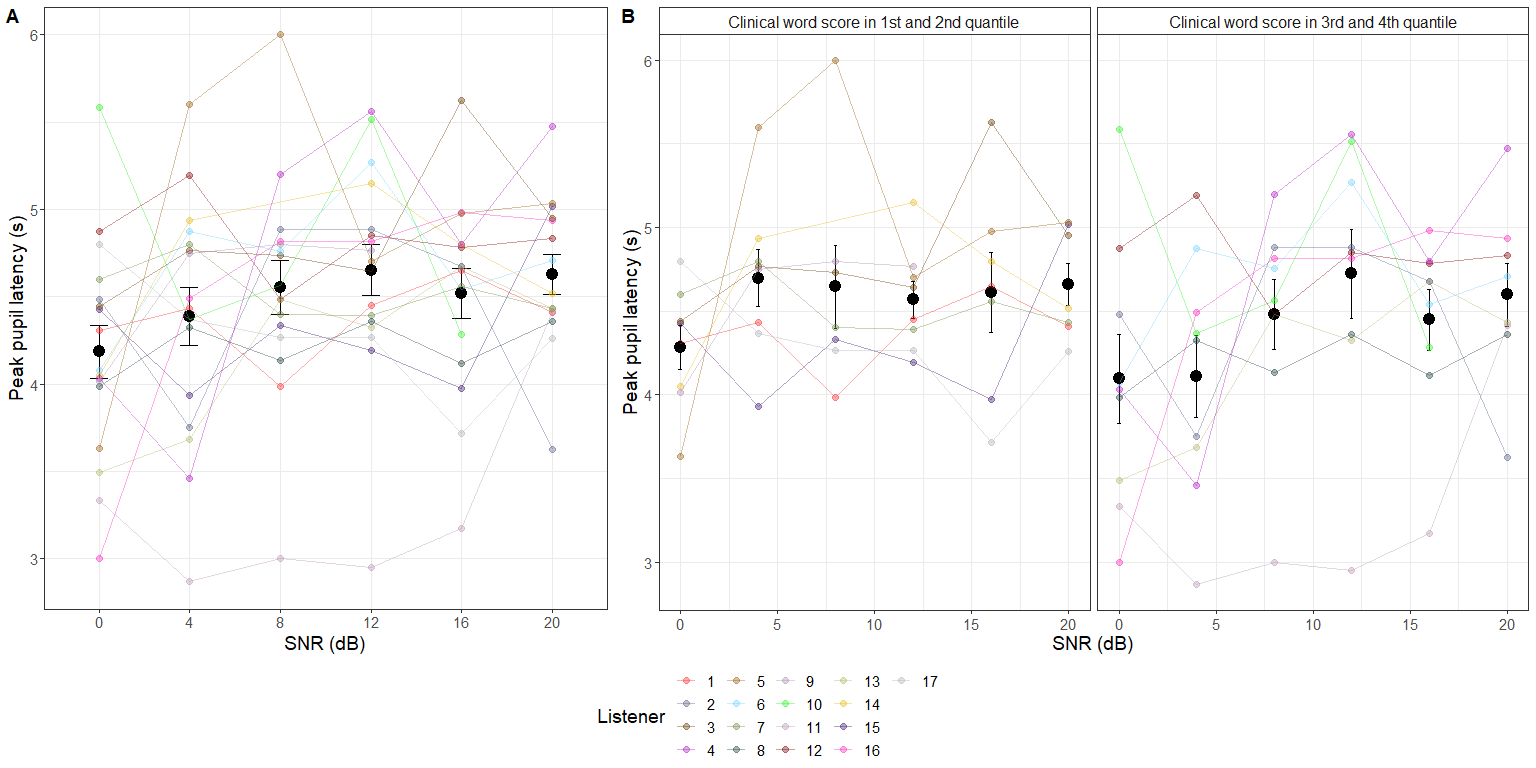  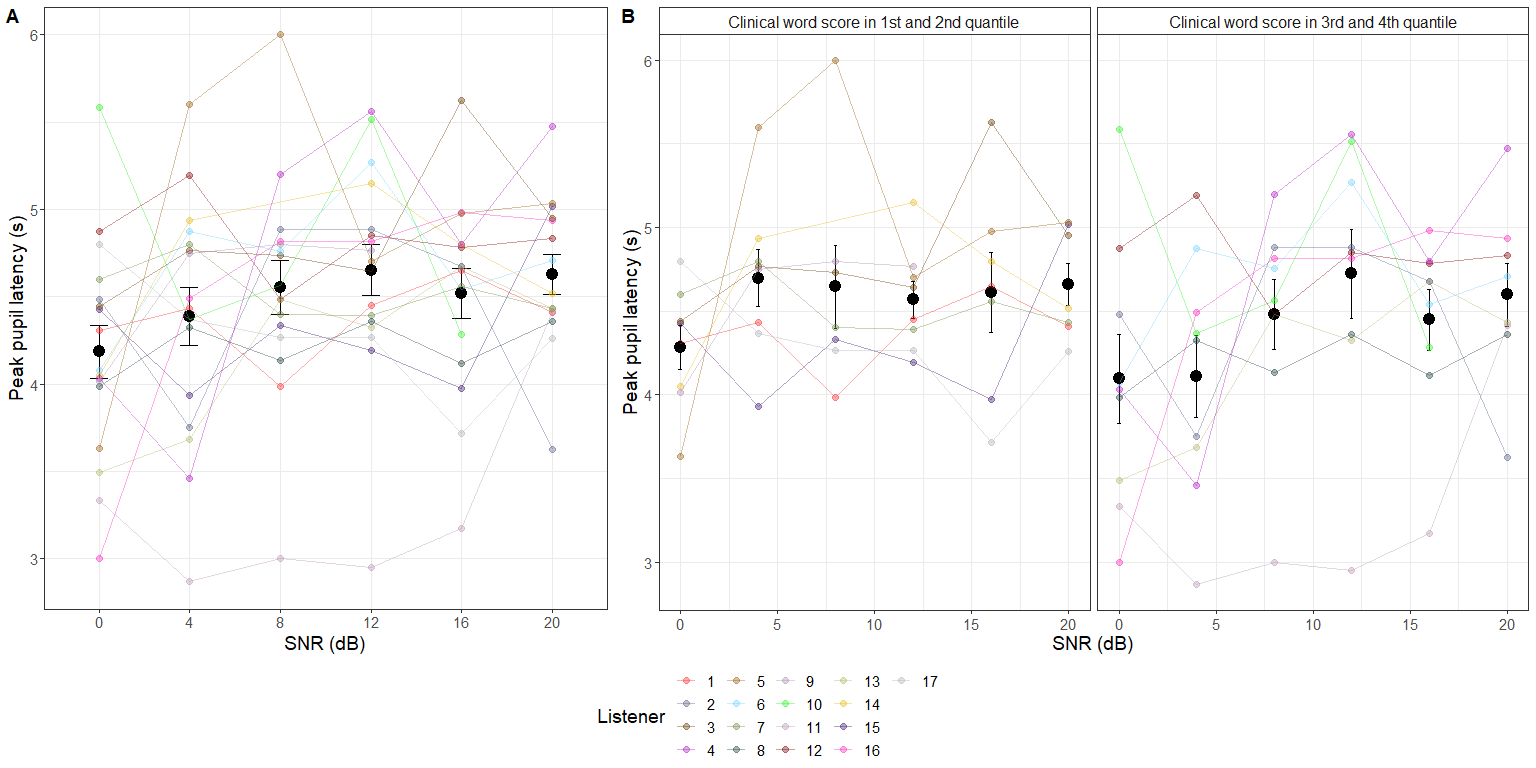  **S2_Fig2:** Psychometric relation between peak pupil latency and SNR, both at a group level (panel A) and split by the median of clinical word recognition scores (panel B). |
| --- |

S2_Fig3 shows the psychometric relation between baseline pupil diameter (measured in arbitrary units standardized by the baseline mean) and SNR (panel A), and a split of CI participants based on the same rule as Figure 4 (panel B).

| 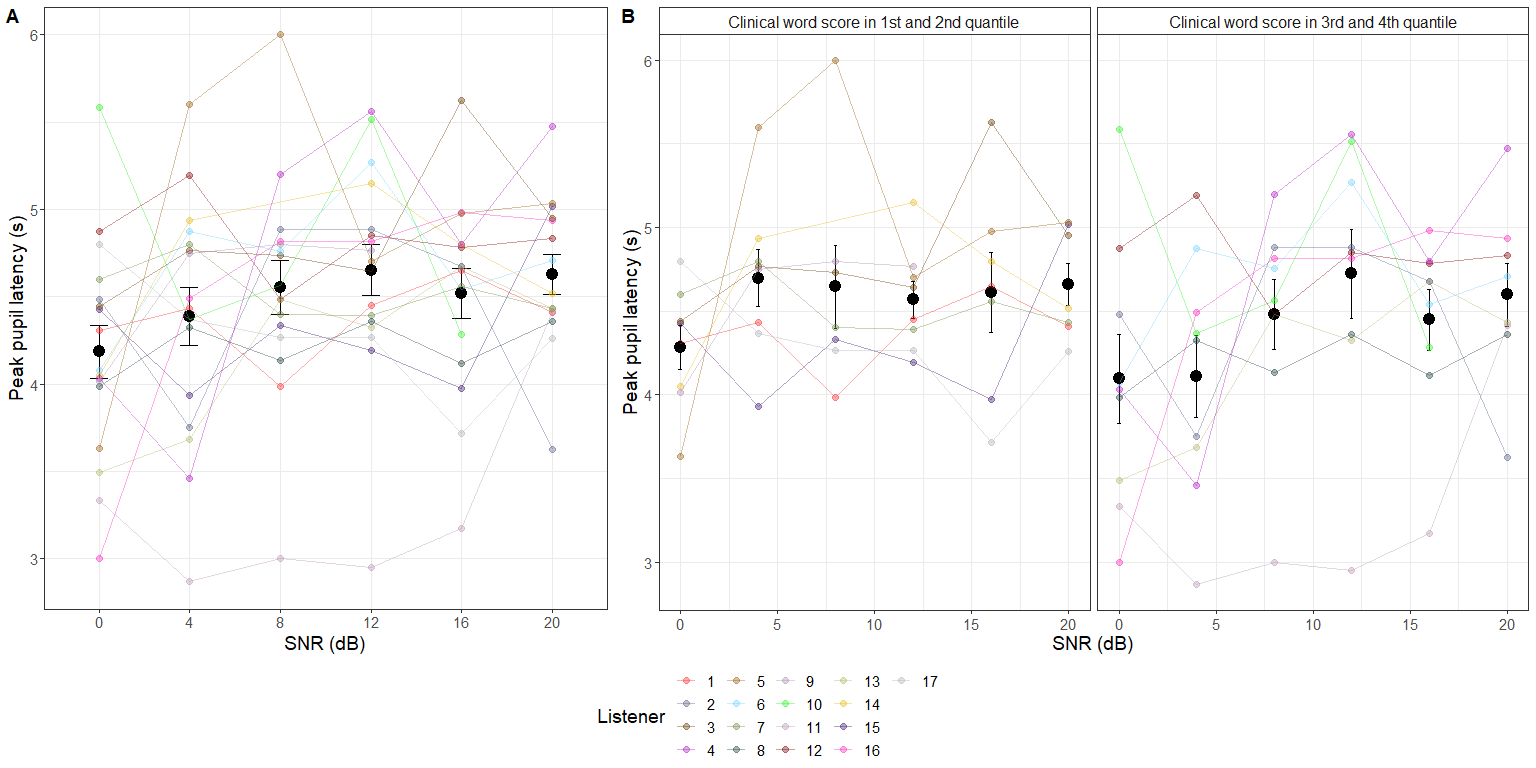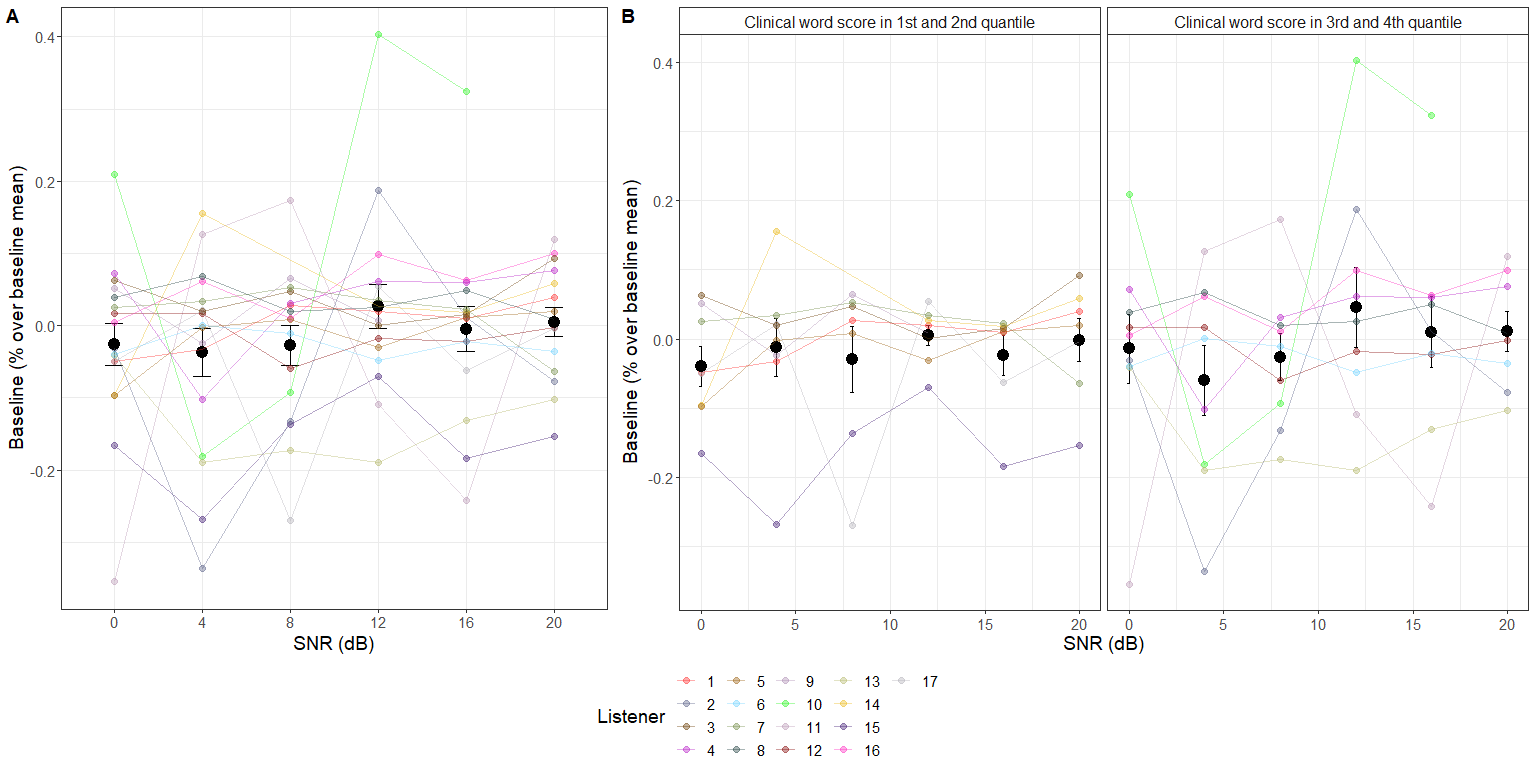  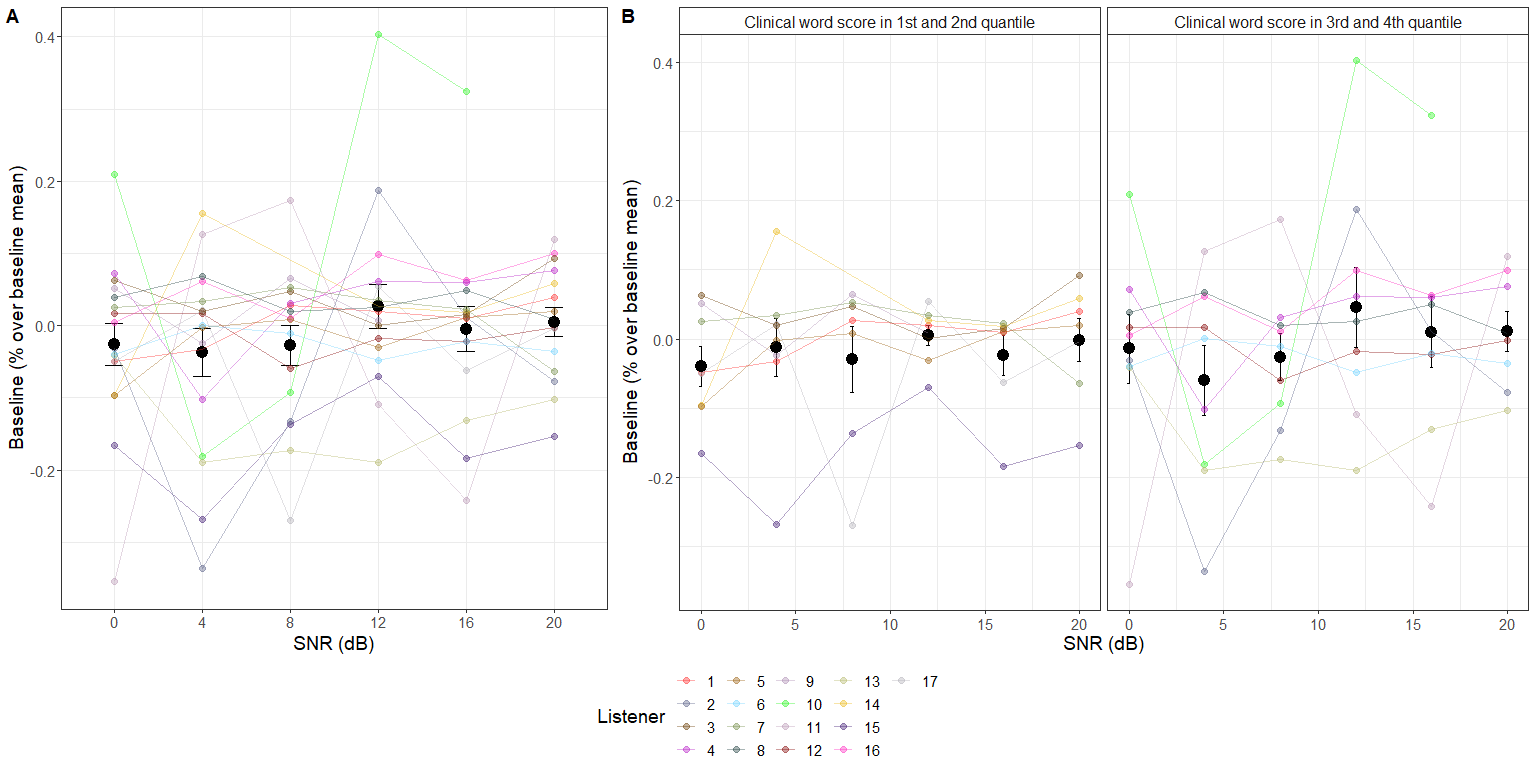  **S2_Fig3:** Psychometric relation between pupil baseline and SNR, both at a group level (panel A) and split by the median of clinical word recognition scores (panel B). |
| --- |
|  |

To conclude, future studies with more CI participants are needed to reveal the relation of different individual factors and hearing outcomes. Compared to PPD, neither baseline nor peak pupil latency is more robust to exhibit the relation between SNR and listening effort. Arguably, the two pupillary measures might relate to other aspects of listening experiences, such as motivation, attention and efficiency.

**
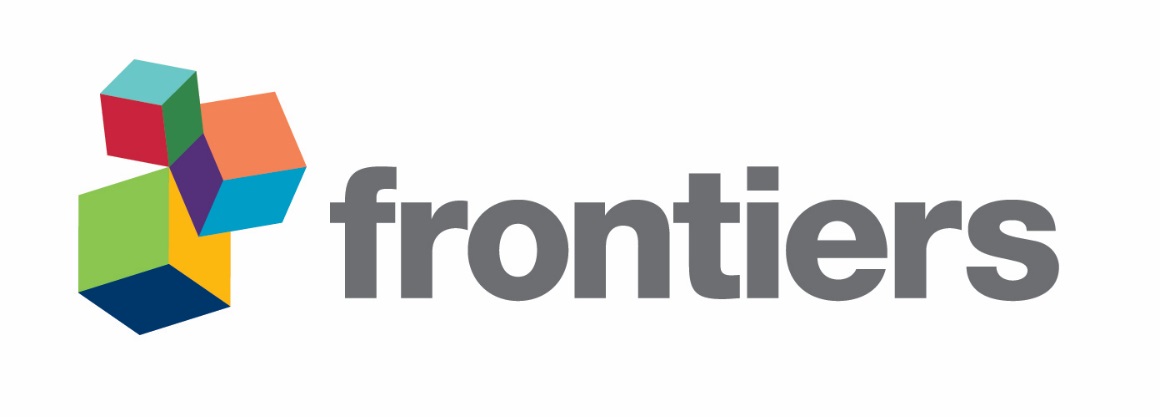
**
