# Supplementary material for: Impact of SNR, peripheral auditory sensitivity, and central cognitive profile on the psychometric relation between pupillary response and speech performance in CI users
Source: Front Neurosci. 2023 Dec 21;17:1307777. doi: 10.3389/fnins.2023.1307777 (PMC10768066; doi:10.3389/fnins.2023.1307777)
Supplement: Supplementary file 3 [file Data_Sheet_3.docx]

Supplementary Material 3

Impact of SNR, peripheral auditory sensitivity, and central cognitive profile on the psychometric relation between pupillary response and speech performance in CI users

Yue Zhang^*^, Amparo Callejón-Leblic, Ana M Picazo-Reina, Sergio B Trejo, Francois Patou, Serafín Sánchez-Gómez

*** Correspondence:** Corresponding Author: yuza@oticonmedical.com

This supplementary material plots similar plot as Figure 4 and Figure 5 panel B to indicate the psychometric relation between SNR and PPD, but only for CI participants who have clinical word recognition in the 1st and 4th quantile. Compared to the analysis in the main body, including only half of the participants for the visualization is less representative of the significant correlation between clinical word recognition and quadratic term of PPD psychometric curve. However, the visualization in this supplementary material can further support the interpretation of the correlation by selecting the CI sub-groups that are more extreme in their clinical word performance. As shown in S3_Fig1, the psychometric relation between PPD and SNR remains similar to that shown in Figure 4 and Figure 5 panel B. This sanity check ensures that the interpretation of the correlation between clinical word recognition and quadratic term is not biased by the group splitting method used in the main paper. But rather, there is a consistent progression of the quadratic term of the PPD psychometric curve, from the lowest to the highest performing CI users.

| 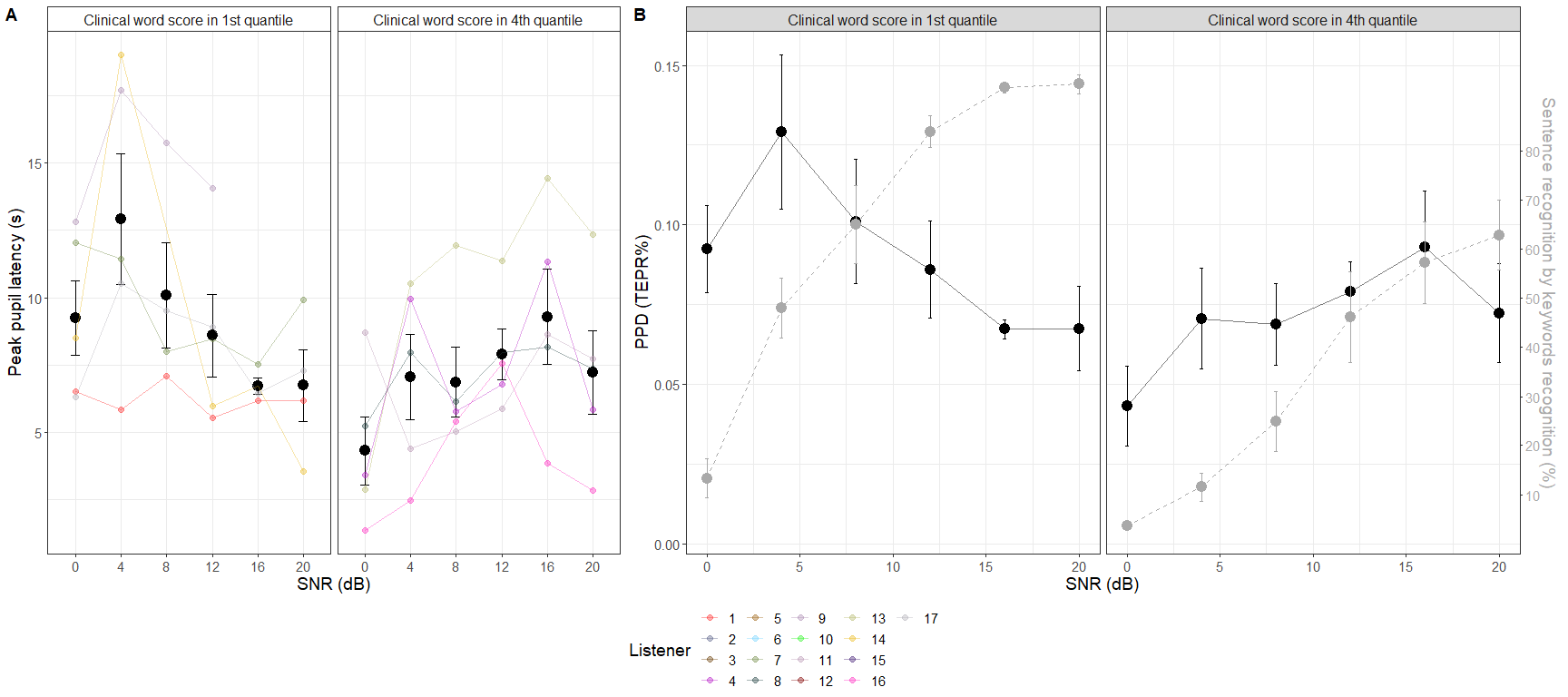  **S3_Fig1:** Psychometric relation between PPD and SNR, split by the 1st and 4th quantile of clinical word recognition scores (panel A). Panel B shows a double-psychometric curve, between PPD, SNR and corresponding sentence recognition scores. |
| --- |

**
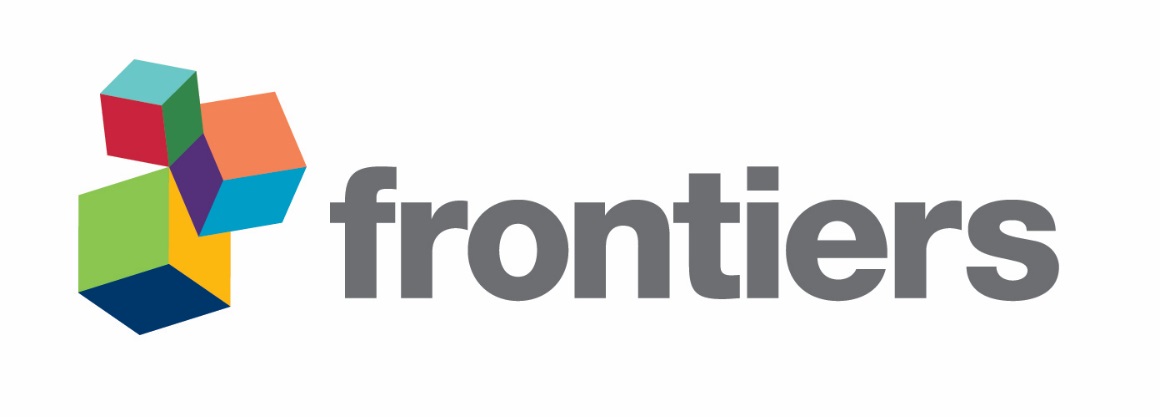
**
